# Stereoselective Conversions of Carbohydrate Anomeric Hydroxyl Group in Basic and Neutral Conditions

**DOI:** 10.3390/molecules30010120

**Published:** 2024-12-31

**Authors:** Monika Khaleri, Qingjiang Li

**Affiliations:** Department of Chemistry, University of Massachusetts Boston, Boston, MA 02125, USA; monika.lnu001@umb.edu

**Keywords:** glycosylation, hemiacetal, stereo control, S*_N_*2, S*_N_*Ar, Mitsunobu, Appel

## Abstract

The rapidly growing glycoscience has boosted the research on the synthesis of glycans and their conjugates, which are centered on the stereoselective formation of glycosidic bonds. Compared to the mainstream acid-promoted glycosylation method that undergoes the S*_N_*1 type mechanism, the basic/neutral conditions give better stereo control via the S*_N_*2 mechanism. Anomeric hydroxyl group transformation, whether to form glycosidic bonds directly or to install a leaving group for later glycosylation, is key to carbohydrate synthesis, and the strategies in the stereo control of these reactions under basic/neutral conditions are summarized in this review. Different stereo control strategies that are applicable to protected or unprotected hemiacetals are discussed, and case-by-case studies of literature reports in the past two decades are included. In addition to surveying literature reports, this review aims at providing insights into the strategic considerations in the development of a stereoselective method for the formation of glycosidic bonds.

## 1. Introduction

Carbohydrates are ubiquitous molecules in nature that function as energy sources and structural components, and are involved in many essential biological processes as signaling molecules. By interacting with protein receptors or glycans, they mediate cell–cell recognition in fertilization, virus/bacteria adhesion, apoptosis, inflammation, immune response, etc. [1,2]. Driven by the significance of carbohydrates and their potential clinical impacts, studies of glycobiology, including the functions, structure, evolution, and interactions with proteins, are booming [3]. However, these studies are often limited by the accessibility of homogeneous carbohydrates with structural diversity, which can increase complexity, thereby imposing great challenges to synthesis. Mimicking the biosynthetic pathways that are executed efficiently by nature, enzymatic synthesis adopts bacterial enzymes to regio- and stereoselectively assemble monosaccharides, but falls short at providing structurally modified glycans catering to the specific request of biological studies. Chemical synthesis, on the other hand, allows structural modification, but is confronted with regio- and stereoselectivity issues, which necessitates sophisticated strategies for protecting group manipulation and for stereo control, which lead to lengthy routes and low efficiency [4,5].

**Scheme 1:**
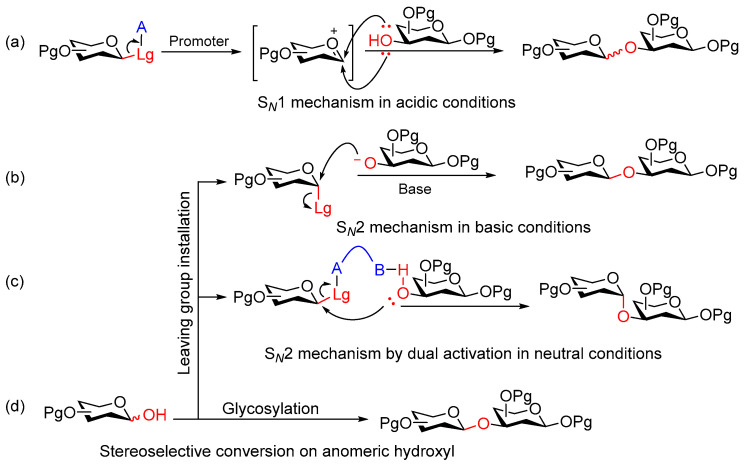
(**a**) S***_N_***1 mechanism in acidic conditions. (**b**) S***_N_***2 mechanism in basic conditions. (**c**) S***_N_***2 mechanism by dual activation in neutral conditions. (**d**) Stereoselective conversion of the anomeric hydroxyl.

The synthesis of complex carbohydrates is essentially the block-wise assembly of monosaccharides via the glycosidic bond, which is an acetal bond connecting the anomeric position of the glycosyl donor to the alcoholic hydroxyl group on the acceptor. The formation of this bond, a process called glycosylation, involves the attack from the weak nucleophilic hydroxyl group on the acceptor to the electrophilic anomeric carbon center of the glycosyl donor, and replaces the leaving group via the mechanism at the borderline of S*_N_*1 and S*_N_*2. In accordance with the low nucleophilicity of the hydroxyl group, a strong electrophile oxocarbenium ion is generated upon activation of the leaving group, typically by Lewis acids. This active intermediate can ensure high yields but requires strict reaction conditions and often results in stereoisomers caused by the attack from either side (Scheme 1a). Realizing the innate limitation of the S*_N_*1 pathway, the recent methodological development in seeking stereoselectivity in glycosylation has shifted to other pathways, such as S*_N_*2, cross-coupling, and radical pathways.

To push the reaction pathway from S*_N_*1 to the stereoselective S*_N_*2 mechanism, several strategies have been crafted. The most intuitive method is to increase the nucleophilicity of hydroxyl groups by deprotonation, generating more nucleophilic oxide ions to attack the carbon with the leaving groups of proper nucleofugality (Scheme 1b). Another strategy involves the simultaneous activation of both the anomeric leaving group on the donor and the hydroxyl group on the acceptor (Scheme 1c). This dual activation of the two functional groups often accompanies the tethering of the donor and acceptor with an organic or metal catalyst [6,7].

The mechanistic foundation for the stereo control in the S*_N_*2 reaction is the backside attack of nucleophiles to facilitate the departure of the leaving group, resulting in inverted configuration at the anomeric carbon center. Therefore, the sole anomer of the donor must be used to afford an optical pure product. This would request the stereo control of the installation of the leaving group at the anomeric hydroxyl group. Evidently, the stereo control of the conversion of the anomeric hydroxyl group, either in the direct construction of the glycosidic bond or the installation of the leaving group (Scheme 1d), holds the key to the real breakthrough in carbohydrate synthesis. Recent progress on such stereoselective conversions has witnessed various new methodologies, including S*_N_*2, S*_N_*Ar, the Mitsunobu reaction, and metal-catalyzed cross-coupling reactions. Most commonly, the alcoholic hydroxyl groups are protected, leaving the hemiacetal hydroxyl group for reaction as a nucleophile. On unprotected sugar, the more acidic anomeric hydroxyl group can be differentiated from alcoholic hydroxyls and selectively reacted based on enhanced nucleophilicity after deprotonation in basic conditions. Direct anomeric functionalization on unprotected/minimal protected sugars has also been realized and applied to the synthesis of biorelevant carbohydrate derivatives.

Stereo control of the direct conversion of anomeric hydroxyl must consider the anomeric effect, which dictates the *α*/*β* ratio of anomeric hydroxyl. Although the cause is still under debate between the n-σ* hyperconjugation hypothesis and n-n repulsion theory, it is undisputable that the lone pair of oxygen in the pyranose ring energetically favors the *α* anomeric hydroxyl group, causing a higher percentage of α anomers over *β*. Normally, this energetic difference is not sufficient for satisfactory control of the stereoselective conversion, but when deprotonated, the negative charge on the anomeric oxygen may enlarge the energy difference of the two anomers and amplify the *α*/*β* ratio. Conversions that can petrify this ratio may give satisfactory stereoselectivity of α-glycosides. The thermodynamically unfavored *β* hydroxyl group, on the other hand, is more nucleophilic due to n-n repulsion. Differentiating the reactivity towards electrophiles, the kinetic product of *β*-glycosides can be obtained selectively.

Methodologies utilizing the nucleophilicity of anomeric hydroxyl or alkoxide ion are normally conducted in basic conditions or natural conditions combined with dual activation. This review intends to survey these methods and summarize the advances in the past two decades to provide insights into future directions. In many cases, the glycosyl donors, especially 1-oxygen substituted glycosyl donors, are generated in situ followed by instant glycosylation in a one-pot method. We will focus on the formation of such donors, since their activation is beyond the scope of this review.

## 2. Conversions of Anomeric Hydroxyl Group on Protected Hemiacetal

### 2.1. Direct Nucleophilic Substitution (S_N_2)

Deprotonation of the anomeric hydroxyl group of a sugar under basic conditions generates strong nucleophiles to attack the electrophilic center of another sugar molecule, displace the leaving group, and form a glycosidic bond by substitution. The anomeric effect renders the *β*-oxide ion a better nucleophile, while the *α*-oxide ion is the predominant species, especially at elevated temperature. During the nucleophilic substitution, factors like the leaving group potency and steric hindrance of the carbon center become critical in determining which anomeric oxide is the prevalent reacting species, thus dictating the anomeric configuration of the products.

**Scheme 2:**
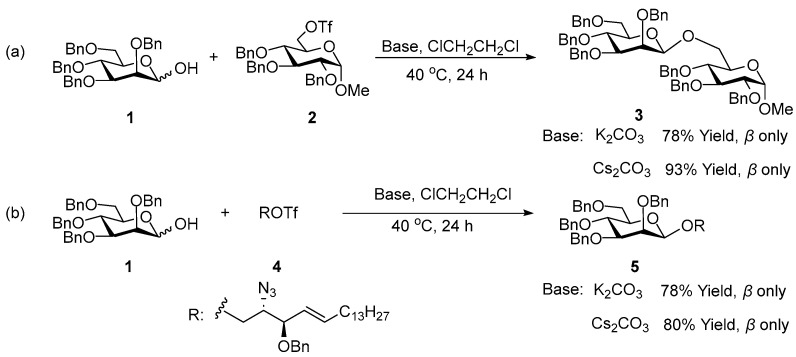
(**a**) Anomeric *O*-alkylation of sugar lactol with primary triflate. (**b**) Synthesis of GSLs.

Many methods have been reported using strong bases like NaH for deprotonation of the anomeric hydroxyl group. Zhu and coworkers developed an effective method for direct and stereoselective synthesis of *β*-mannopyranosides via anomeric *O*-alkylation using milder bases like K_2_CO_3_ and Cs_2_CO_3_ [8]. When sugar lactol **1** was reacted with C6-primary triflate **2** in the presence of K_2_CO_3_ or Cs_2_CO_3_, *β*-linked disaccharide **3** was afforded in good yield and with excellent anomeric selectivity (Scheme 2a). It is hypothesized that a kinetic anomeric effect and metal chelation work in concert to govern the stereoselectivity of the reaction. For the effective alkylation using Cs_2_CO_3_ as a base, the lactol donors must have a conformationally flexible C6 oxygen atom to chelate with a cesium ion. C2 oxygen has no significant effect on stereoselectivity, indicating the lack of neighboring group participation in the reaction pathway.

The studies indicated that K_2_CO_3_ is less effective for stereoselective anomeric alkylation of partially protected mannose with triflate of secondary alcohol, whereas Cs_2_CO_3_ tolerates both primary and secondary triflate electrophiles. Also, reactions involving K_2_CO_3_ afforded desired products in slightly lower yields as compared to Cs_2_CO_3_ (Scheme 2a). Nonetheless, K_2_CO_3_ was chosen for the following studies because of its milder basicity and economic reasons.

This method has been utilized in the synthesis of various glycosphingolipids (GSLs). GSL syntheses predominantly rely on acid-catalyzed glycosylation to form the glycosidic bond between the sugar moiety and sphingosine derivatives. This K_2_CO_3_-mediated method demonstrated excellent stereoselectivity in the coupling of carbohydrate **1** and sphingosine triflate derivatives **4** by anomeric *O*-alkylation for GSL synthesis.

**Scheme 3:**
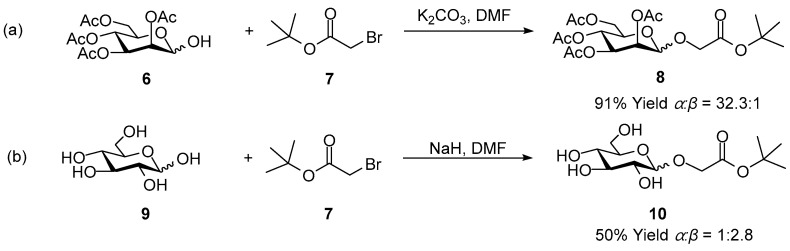
(**a**) Synthesis of glycosyl esters using protected sugars. (**b**) Synthesis of glycosyl esters using unprotected sugars.

When changed from triflate to bromide, the leaving group potency decreases and the stereoselectivity is compromised. Queneau et al. reported the synthesis of mono- and disaccharidic carboxymethyl glycoside lactones (CMGLs) via glucosyl ester intermediates prepared by alkylation of hemiacetals [9]. As shown in Scheme 3, hemiacetal **6** was deprotonated by K_2_CO_3_ and replaced the bromide in *tert*-butyl bromoacetate **7**. The glycosyl esters were obtained as a mixture of anomers with predominant *α*-anomer in good yield. Different mono- and disaccharides were applied to this methodology, which afforded corresponding esters with high *α*-selectivity. With unprotected sugars, the reaction was carried out in the presence of NaH (Scheme 3b). An interesting finding is that glucose **9** provided esters **10** with high *β*-selectivity, whereas galactose afforded *α*-furanoside and *N*-acetyl and glucosamine afforded *α*-pyranoside as the major product.

### 2.2. Formation of Imidates by Addition to π-Bond

Glycosyl imidates are among the most popular leaving groups in glycosylation due to their facile synthesis, easy and versatile activation, and high efficiency in glycosylation. Since their introduction by Schmidt et al. in 1980, glycosyl trichloroacetimidates have been extensively utilized in carbohydrate synthesis [10]. To suppress the side reaction caused by the nucleophilic attack from the nitrogen atom, *N*-phenyl trifluoroacetimidate was later introduced by the Yu group [11]. Typically, nitriles or ketenimines with electron-withdrawing substituents were used to yield imidates by reacting with anomeric hydroxyl. The anomeric configuration of trichloroacetimidates may be controlled; however, different anomers have little effects on the stereoselectivity of subsequent glycosylation, which is generally believed to undergo S*_N_*1 mechanism with imidate donors.

**Scheme 4:**
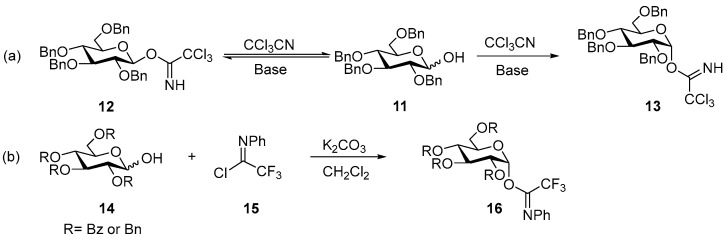
(**a**) Synthesis of glycosyl trichloroacetimidates. (**b**) Synthesis of glycosyl trifluoroacetimidates.

Addition of a hemiacetal to trichloroacetonitrile, in the presence of either inorganic (NaH, K_2_CO_3_, Cs_2_CO_3_, etc.) or organic (DBU) bases, stereoselectively gives glycosyl trichloroacetimidates. Deprotonation of the anomeric hydroxyl group takes place under basic conditions giving a highly nucleophilic oxide ion, which attacks the electrophilic center of trichloroacetonitrile and gives glycosyl imidates. The treatment of 2,3,4,6-tetra-O-benzyl-D-glucose **11** with trichloroacetonitrile predominantly afforded *β-*trichloroacetimidate **12**. However, this product undergoes base-catalyzed anomerization to give *α*-trichloroacetimidate **13** which is favored thermodynamically due to the anomeric effect (Scheme 4a). Therefore, both *α* and *β* anomers can be obtained in pure form and high yield. Synthesis of glycosyl (*N*-phenyl)trifluoracetimidates was achieved by an addition–elimination reaction. The treatment of acetyl-, benzoyl-, and benzyl-protected hemiacetals **14** with *N*-phenyl trifluoroacetimidoyl chloride **15** in the presence of K_2_CO_3_ provided corresponding *α*-glycosyl trifluoroacetimidates **16** predominantly due to the anomeric effect (Scheme 4b). In fructofuranosides, where the synthesis of the trichloroacetimidate was difficult, using *N*-phenyltrifluoroacetimidate was an effective alternative.

**Scheme 5:**
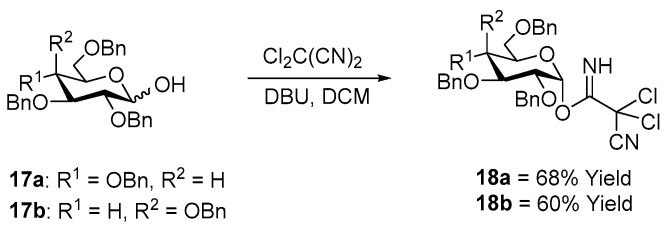
Synthesis of dichloro-cyanoacetimidate glycosyl donor.

The Schmidt group also prepared glycosyl donors with dichloro-cyanoacetimidate as the leaving group [12]. The treatment of gluco- and galactopyranose **17** with dichloromalonitrile in the presence of 1,8-Diazabicyclo[5.4.0]undec-7-ene (DBU) afforded *O*-glycosyl dichloro-cyanoacetimidate **18** in good yields with *α-*selectivity (Scheme 5).

**Scheme 6:**
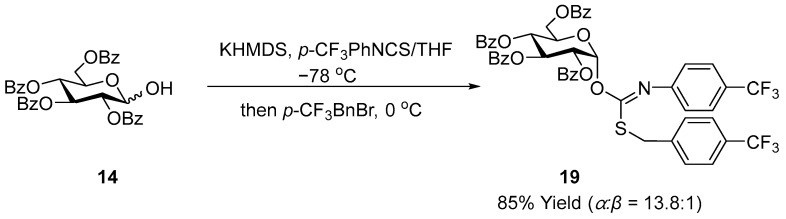
Formation of *p*-trifluoromethylbenzylthio-*p*-trifluoromethylphenyl formimidate glycosyl donor.

The Mukaiyama group further explored new and effective glycosyl donors with a leaving group that had two activation sites, i.e., imidate and thio linkages, within the same leaving group [13]. The treatment of the anomeric hydroxyl group of 2,3,4,6-tetra-*O*-benzoyl-D-glucopyranose **14** with *p*-trifluoromethylphenyl isothiocyanate followed by treatment with *p*-trifluoromethylbenzyl bromide under basic conditions afforded 2,3,4,6-Tetra-*O*-benzoyl-*α*-D-glucopyranosyl *p*-trifluoromethylbenzylthio-*p*-trifluoromethylphenyl formimidate **19** with high *α*-stereoselectivity (Scheme 6).

**Scheme 7:**
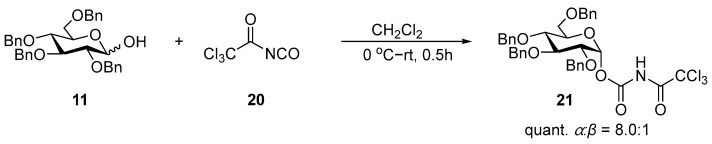
Synthesis of *N*-trichloroacetyl carbamate glycosyl donor.

To develop a new type of glycosyl donor with *N*-trichloroacetyl carbamate as a leaving group, Omura and colleagues reacted benzyl-protected glucopyranose **11** with trichloroacetyl isocyanate **20** in dry CH_2_Cl_2_ [14]. The reaction quantitively afforded the desired donors **21** as a mixture of anomers with good *α*-selectivity (Scheme 7).

### 2.3. Esterification of Anomeric Hydroxyl by Addition-Elimination

Esterification is among the most studied reactions and can be achieved in numerous conditions with a large variety of esterification reagents. The great variety of esterification methodologies offered carbohydrate chemists opportunities to explore glycosyl esters as donors in glycosylation. Carboxylate, phosphate, and sulfate are among the most studied esters and will be elaborated in the following section.

#### 2.3.1. Formation of Carboxylate Ester

**Scheme 8:**
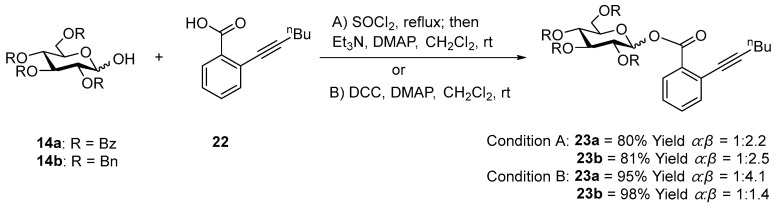
Synthesis of *O*-alkynylbenzoates.

Glycosyl esters are promising glycosyl donors for oligosaccharide synthesis and can be prepared by direct condensation of carboxylic acids with hemiacetal [15]. Simple glycosyl esters like acetates and benzoates have mediocre reactivities and typically require strong acid or stoichiometric promoters for activation [16]. However, when combined with proper functional groups, they can be efficiently activated. Donors with modified ester leaving groups have been reported in recent years, among which the *O*-alkynylbenzoates reported by Yu et al. stood out for its easy activation due to the presence of triple bonds [17]. Two different methods were examined for the condensation of sugar lactols **14** with *o*-hexynylbenzoic acid **22** affording glycosyl *o*-hexynylbenzoates **23** (Scheme 8). In the first method, the anomeric hydroxyl group is converted to sulfonate while fixing the configuration for displacement by the carboxylate ion via S*_N_*2 mechanism. The second method employed *N*, *N*′-Dicyclohexylcarbodiimide (DCC) as the coupling reagent. Depending on the type of lactols and reaction conditions, a varied ratio of *α:β* anomers was obtained with the kinetic product *β-*anomer as the major isomer. The anomeric mixtures were directly used for further glycosylation reaction without any separation.

**Scheme 9:**
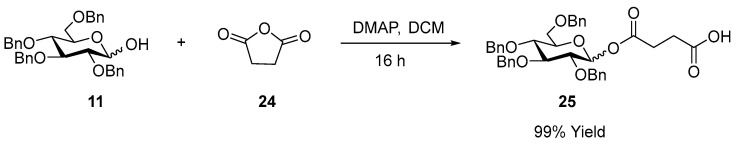
Synthesis of succinyl benzyl glucose.

The Huang group developed a glycosylated Paclitaxel (PTX) prodrug with anti-cancer activity [18]. The coupling of the glucose unit with the PTX prodrug (SG-PTX) by glycosylation involved the formation of glucosyl ester **25**. The treatment of benzyl-protected glucose **11** with succinic anhydride **24** in the presence of DMAP afforded monoester **25** in excellent yield (Scheme 9). The reaction involved deprotonation of anomeric OH in basic conditions and the resulting oxide nucleophile attacked one of the carbonyl groups to open the anhydride ring. Double glucose units were also glycosylated with the PTX prodrug (DG-PTX) using the same reaction conditions. The selectivity of the products was not given.

**Scheme 10:**
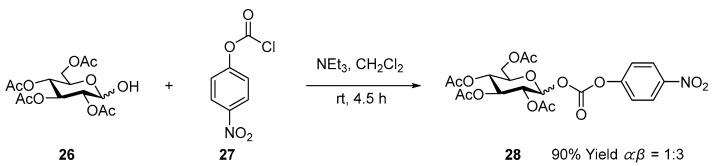
Synthesis of 4-nitrophenyl glycosyl carbonate.

In 2005, the Wang group synthesized a glycosyl carbonate by reacting hemiacetal **26** with 4-nitrophenyl chloroformate **27** in the presence of triethylamine [19]. The desired glycosyl carbonate **28** was obtained as a mixture of anomers with high *β*-selectivity (Scheme 10). This glycosyl carbonate was converted to a carbamate bearing 3-morpholinosydnonimine (SIN-1), which was used to deliver nitric oxide by glycosidase-mediated cleavage. Glucose, galactose, and *N*-acetylneuraminic acid were all applicable to this methodology. In 2016, the Wittmann group also utilized these glycosyl carbonates as precursors to generate carbamate linkage in the synthesis of Neoglycopeptides [20].

#### 2.3.2. Formation of Phosphate

Glycosyl phosphate donors, normally with protecting groups like phenyl, benzyl, or *n*-butyl, are widely used in glycosylation. Interestingly, phosphates are nature’s favorite leaving groups in the biosynthesis of protein, DNA, and carbohydrates, because of their kinetic stability and Lewis base character [21]. As a glycosyl donor in oligosaccharide synthesis, glycosyl phosphate can be activated by Lewis acids to stereoselectively form glycosidic bonds. They can be prepared by direct phosphorylation of glycosyl hemiacetal with anhydride or via phosphite followed by oxidation [22].

**Scheme 11:**
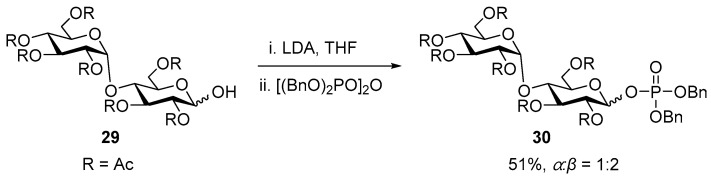
Synthesis of acetyl-protected D-maltosyl Phosphate in presence of LDA.

In 2011, the Bornemann group reported a method to synthesize a D-maltose 1-phosphate donor by nucleophilic substitution and it was used to evaluate the activity of GlgE, a maltosyltransferase involved in bacterial-glucan biosynthesis and a genetically validated antituberculosis target [23]. The first step of synthesis was the preparation of acetyl-protected D-maltosyl Phosphate **30** by treatment of *O*-acetyl-D-maltose **29** with tetrabenzyl pyrophosphate in the presence of Lithium diisopropylamide (LDA). The desired phosphate was obtained in moderate yield as a mixture of anomers (Scheme 11), which was further deprotected to yield a D-maltose 1-phosphate donor.

**Scheme 12:**
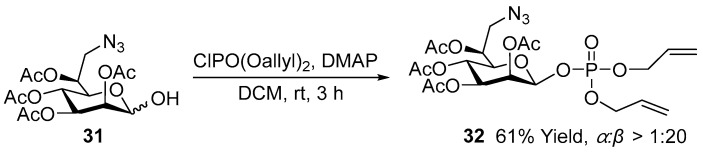
Synthesis of heptoside-1-*β*-phosphate.

In 2021, the Vincent group reported the synthesis of ADP-7-Azido-heptose, a bacterial metabolite found in Gram-negative bacteria, through a heptoside-1-*β*-phosphate intermediate prepared by stereoselective phosphorylation of the lactol moiety [24]. The 7-Azido-D-manno-heptopyranose hemiacetal **31** was treated with diallyl chlorophosphate in the presence of DMAP, affording heptoside phosphate **32** in moderate yields with excellent *β-*selectivity (Scheme 12). Diphenyl chlorophosphate was also used as a phosphorylating agent but only gave very low selectivity.

Sauvageau and coworkers reported a method for the phosphorylation of hemiacetals using same above-mentioned conditions, during the study of 7-*O*-modification of D-glycero-*β-*D-mannoheptose phosphate [25]. However, they used diphenyl cholorophophate as the phosphorylating agent, which provided low-to-moderate yields with low selectivity.

**Scheme 13:**
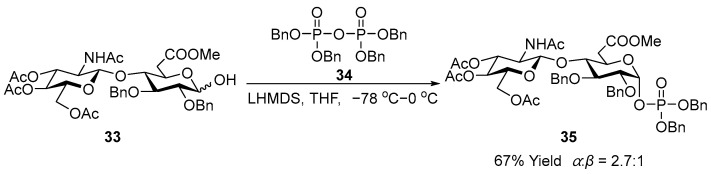
Synthesis of GlcNAcβ(1→4)GlcUA-1-phosphate.

Kisko and coworkers reported a method to synthesize GlcNAcβ(1→4)GlcUA–UDP, a disaccharide donor for the enzymatic synthesis of hyaluronic acid [26]. It started with the phosphorylation of GlcNAcβ(1→4)GlcUA hemiacetal at the anomeric position in basic conditions by nucleophilic substitution. When disaccharide unit **33** was treated with lithium hexamethyldisilazide (LHMDS), the lithium alkoxide of anomeric OH was formed and attacked tetrabenzylpyrophosphate **34** to afford GlcNAcβ(1→4)GlcUA-1-phosphate **36** derivative with *α*-selectivity (Scheme 13).

**Scheme 14:**
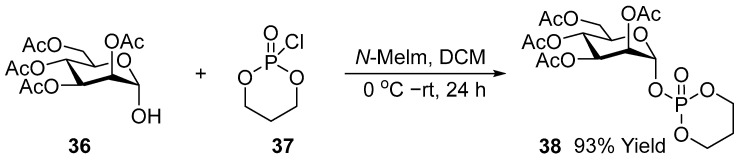
Synthesis of glycoside phosphate.

In the synthesis of an analogue of the hexasaccharide antigen for the construction of a dengue vaccine, Singh et al. extensively used the phosphate donor synthesized by phosphorylation of hemiacetal [27]. The treatment of *α*-anomer of acetyl-protected mannose **36** with propane-1,3-diyldioxyphosphoryl chloride **37** in the presence of *N*-Methylimidazole (*N*-Melm) afforded the desired phosphate glycoside **38** with *α*-selectivity in excellent yield (Scheme 14). Various monosaccharide units and trisaccharide were all converted to the phosphate en route to the synthetic target of hexasaccharide analogue.

**Scheme 15:**
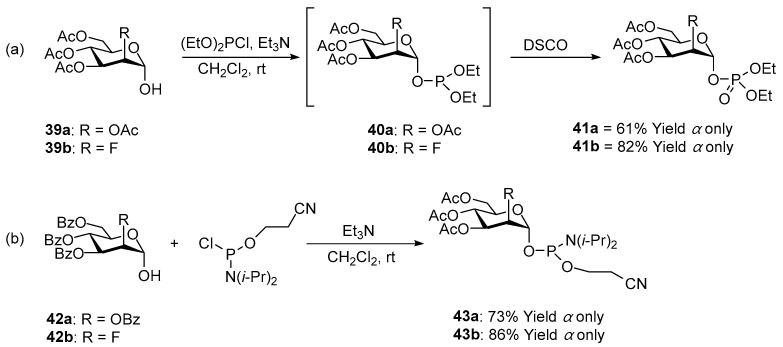
(**a**) Synthesis of phosphate ester derivatives using diethyl chlorophosphite. (**b**) Synthesis of Mannosyl phosphate using 2-cyanoethyl-*N*,*N*-diisopropylchlorophosphoramidite as a phosphorylating agent.

The *α*-stereoselectivity of phosphorylation is independent of the C2 substitution, as demonstrated in the synthesis of 2-Deoxy-2-fluoromannosyl phosphotriester and phosphodiester derivatives reported by the Wada group [28]. As shown in Scheme 15, phosphotriester analogs **40** varying in their C2 substituent were synthesized by treatment of protected mannose **39** with diethyl chlorophosphite in the presence of triethylamine. Subsequent oxidation of these intermediates with dichlorocamphorylsulfonyl oxaziridine (DCSO) afforded glycosyl phosphates **41** as *α*-anomers in good yield (Scheme 15a). 2-cyanoethyl-*N*,*N*-diisopropylchlorophosphoramidite was also used as a phosphorylating agent to synthesize Mannosyl phosphate with excellent *α*-stereoselectivity (Scheme 15b).

**Scheme 16:**
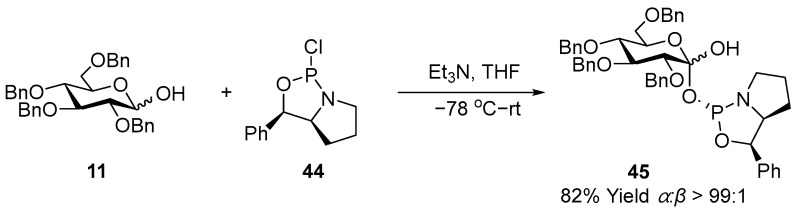
Synthesis of glycosyl phosphate with the help of oxzaphospholine derivatives.

Phosphitylation of anomeric hydroxyl is also *α*-selective, as observed by Wada et al. in the synthesis of **45**. The treatment of protected hemiacetals with 2-chloro-1,3,2-oxazaphospholidine **44** in the presence of triethylamine afforded the desired glycosides in good yields (Scheme 16). Glucopyranosyl and mannopyranosyl derivatives both predominantly gave *α*-anomer [29].

**Scheme 17:**
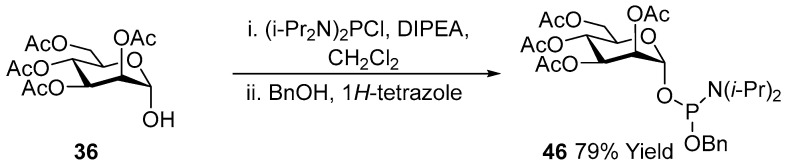
Synthesis of phosphoramidite derivatives.

To synthesize various fragments of parasitic proteophosphoglycans, Boons and coworkers reacted the hydroxyl group of various amino acids and peptides with *α*-mannosyl and *α*-*N*-acetyl-glucosamine phosphoramidite [30]. The phosphate derivatives used were prepared stereoselectively by the treatment of D-mannose **36** with *N*,*N*-diisopropyl chlorophosphoamidite in the presence of DIPEA followed by substitution with benzyl alcohol. The *α*-glycosyl phosphoramidite intermediates **46** (Scheme 17) obtained from this reaction were reacted with a serine derivative to afford the expected proteophosphoglycans.

#### 2.3.3. Formation of Sulfate and Sulfoxide

To push the reaction mechanism towards S*_N_*2 for stereo control in glycosylation, the leaving group should have proper nucleofugality that favors the bimolecular nucleophilic substitution. Sulfonate is a leaving group well known to facilitate S*_N_*2 reaction and warranted research effort on converting anomeric hydroxyl to sulfonate by reacting with sulfonyl halide or anhydride in the presence of a base. It should be noted that sulfates with strong electron-withdrawing groups such as the trifluoromethyl group are exceptional leaving groups, but often favor the S*_N_*1 pathway, and are thus improper as a leaving group in glycosylation.

The Bennett group reported a stereo-inverting process by examining the factors that favor the S*_N_*2 mechanism, such as converting anomeric hydroxyl into alkoxide for enhanced nucleophilicity, installation of a leaving group with proper nucleofugality, and usage of polar aprotic solvent [31]. Sulfonate was installed and subsequently replaced by the alkoxide nucleophile on the glycosyl acceptor. The anomeric hydroxyl was deprotonated with a strong base and the negative charge amplifies the anomeric effect to give predominantly *α*-alkoxide, which was converted to sulfonate. The *α*-sulfonate donor was attacked by the alkoxide on the acceptor via the stereo-inverting S*_N_*2 pathway to afford *β*-glycosides.

**Scheme 18:**
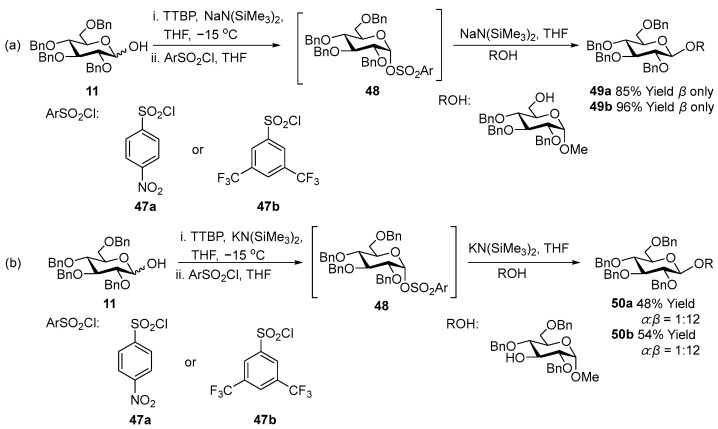
(**a**) Glycosylation of glycosyl sulfonate via S*_N_*2 using primary acceptors. (**b**) Glycosylation of glycosyl sulfonate using secondary acceptors.

As shown in Scheme 18a, the method generated the sulfonate donor **48** in situ by deprotonating the hemiacetal sugar **11** and reacting with sulfonylating agent **47**. The mechanistic study confirmed that the stereoselectivity of the reaction is caused by the stereo-inverting and concerted reaction pathway.

The reaction conditions used were compatible with both acid- and base-sensitive protecting groups that are commonly used in oligosaccharide synthesis, such as 2-naphthylmethyl (Nap), benzoate (Bz), acetate (Ac), and triisopropylsilyl (TIPS) ether. When acceptors with primary alcohol were used, the desired S*_N_*2 pathway dominates and gives high *β*-selectivity regardless of arylsulfonate substitution on the donors (Scheme 18a). However, with less-reactive secondary acceptors, selectivity between the desired S*_N_*2 pathway and the undesired S*_N_*1 pathway undermined the stereoselectivity and the choice of arylsulfonate with proper reactivity and stability becomes critical (Scheme 18b). The electron-rich, more stable arylsulfonates lead to higher selectivity in the reactions.

**Scheme 19:**
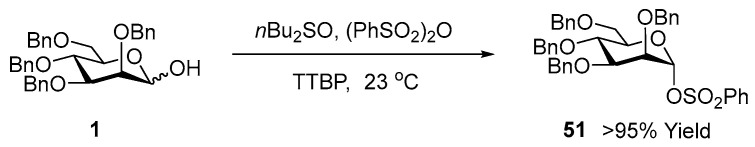
Formation of *α*-mannosyl sulfonate.

Gin and colleagues reported a method for sulfonylation of hemiacetals by sulfonic anhydride in the presence of sulfoxide catalyst [32]. When *O*-benzyl-D-mannopyranose **1** was treated with benzenesulfonic anhydride (PhSO_2_)_2_O in combination with a di-*n*-butyl sulfoxide (*n*Bu_2_SO) catalyst in the presence of 2,4,6-tri-*tert*-butylpyridine (TTBP), *α*-mannosyl sulfonate **51** was formed in excellent yield (Scheme 19). The reaction mechanism was proposed to be initiated by the attack of a sulfoxide catalyst to sulfonic anhydride, generating a glycosyl oxosulfonium ion, which may react with the anomeric hydroxyl and delivers the sulfonyl group to the hemiacetal in two steps. The resulting glycosyl sulfate was used as a donor in glycosylation without purification.

**Scheme 20:**
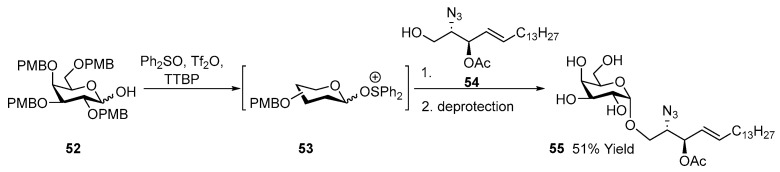
Formation of α-psychosine.

Savage and coworkers reported a method to synthesize pyschosine variants, which are antigens for natural killer T cells [33]. The treatment of protected galactose **52** with diphenyl sulfoxide, Tf_2_O, and 2,4,6-tri-*tert*-butylpyrimidine (TTBP), followed by reaction with sphingosine variant **54**, afforded **55**, which was subjected to deprotection and reduction to give glycolipid alpha-psychosine (Scheme 20). Similarly, *α*-Glc-palmitoylglycerol and different alpha-linked glycolipids were synthesized using this methodology. The beta-linked glycolipids were synthesized using *α*-glycosyl trifluoroacetimidates donors, which were then activated by Trimethylsilyl trifluoromethanesulfonate (TMSOTf) to afford the desired beta glycolipids.

A similar reaction mechanism as above, sulfation, which involves the generation of an oxosulfonium glycosyl donor in situ by reacting with diphenyl sulfoxide and triflic anhydride, can be postulated. Instead of forming glycosyl sulfate, the highly reactive sulfonium is directly attacked by a lipid acceptor, giving rise to the desired glycoside. Although the stereoselectivity in the conversion of anomeric hydroxyl could not be recorded, the final product was obtained as α-configuration.

### 2.4. Esterification Assisted by Coupling Reagents

As previously mentioned, glycosyl ester often bears other functional groups to facilitate the activation in mild conditions for glycosylation. These functional groups may not be compatible with carbonyl halide or anhydride. Therefore, esterification of the anomeric hydroxyl group using carboxylic acids, which are widely commercially available, in mild conditions, is desired. Coupling reagents such as carbodiimide can convert carboxylic acids into active esters, which can be attached by a hydroxyl group to form ester. These methods typically tolerate many functional groups and are performed in mild basic conditions, allowing the control of the anomeric configuration by kinetic or thermodynamic factors.

**Scheme 21:**
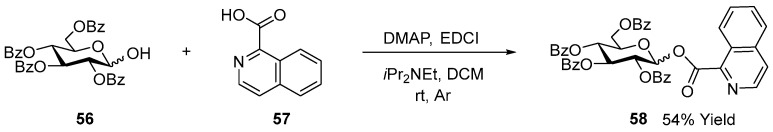
Formation of IQC esters.

Pyridine-2-carboxylates, or picolinic esters as leaving groups on armed glycosyl donors, yielded a low quantity of glycosylated products with a mixture of anomers because these donors were difficult to activate [34]. The Tang group found that disarmed picolinic esters could be activated by metal promoters that not only weakened the anomeric C-O bond, but also activated the carbonyl group towards nucleophilic attack. A significant amount of transesterification byproduct was also observed along with the desired products. Then they added a bulky substituent on the 3-position of picolinic ester, which can block the attack of nucleophiles on the carbonyl group during glycosylation and can minimize the formation of byproducts. Picolinic acid with a 3-substituent, isoquinoline-1-carboxylic acid **58** was used to prepare isoquinoline-1-carboxylate (IQC) esters [35]. These esters were far more reactive than picolinic ester donors. The treatment of hemiacetal sugar **56** with commercially available isoquinoline-1-carboxylic acid **57** in the presence of DMAP, EDCI, and *i*Pr_2_NEt in DCM afforded anomeric isoquinoline-1-carboxylate esters **58** with *α/β*-anomers (Scheme 21).

**Scheme 22:**
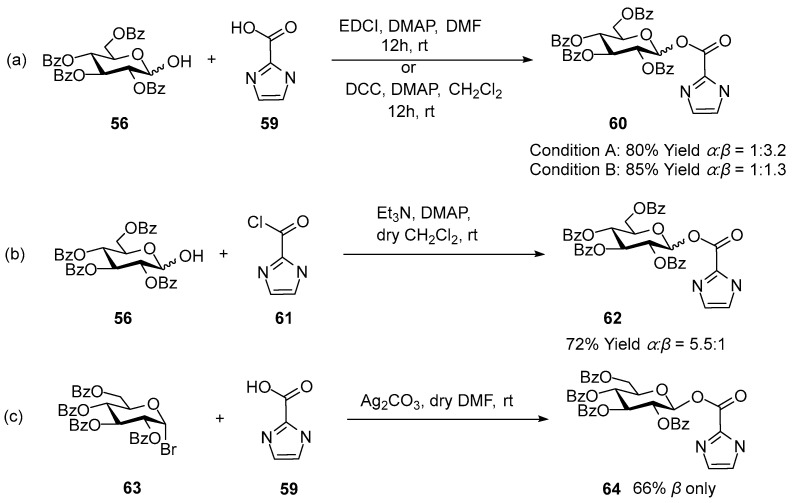
(**a**) Synthesis of glycosyl donors by using 1-methyl-2-imidazolecarboxylic acid. (**b**) Synthesis of Glycosyl Donors by using 1-methyl-1-H-imidazole-2-carboxylic acid chloride. (**c**) Synthesis of pure *β*-anomer of glycosyl 1-methylimidazole-2-carboxylate.

Yu et al. synthesized glycosyl esters bearing an imidazole ring, which proved to be a better leaving group [36]. To obtain glycosyl 1-methylimidazole-2-carboxylates **60**, several methods were examined. Firstly, condensation of 2,3,4,6-tetra-*O*-benzoyl-D-glucopyranose **56** and 1-methyl-2-imidazolecarboxylic acid **59** was tested using either EDCI or DCC as a coupling reagent and DMAP as a base. While both conditions effectively gave the desired product in comparable yields, EDCI was superior in providing better *β*-selectivity. (Scheme 22a). Benzoylated glycosyl hemiacetals, including glucopyranosyl, mannopyranosyl, rhamnopyranosyl, xylopyranosyl, ribopyranosyl, ribofuranosyl, and glucopyranosyl, all were successfully applied to this protocol and delivered the desired glycosyl 1-methylimidazole-2-carboxylates in satisfactory yields. When 1-methyl-1-H-imidazole-2-carboxylic acid chloride **61** was used to react with glycosyl hemiacetal in the presence of Et_3_N and DMAP, product **62** with predominant *α*-anomer was obtained (Scheme 22b). Additionally, pure *β*-anomer **64** was prepared from glucosyl bromide **63** and acid in the presence of Ag_2_CO_3_ in dry DMF (Scheme 22c).

**Scheme 23:**
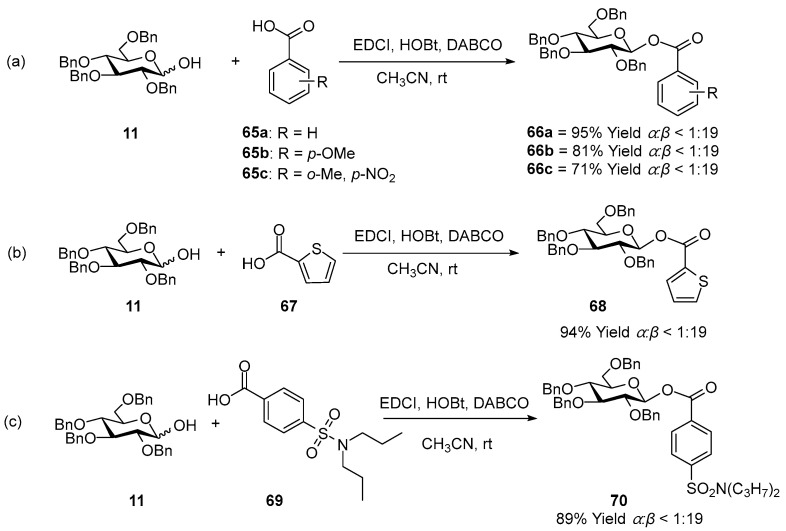
(**a**) Synthesis of *β*-glycosyl esters via HOBt acyl transfer reagent. (**b**) Synthesis of *β*-glycosyl esters using heteroaromatic carboxylic acids. (**c**) Coupling of Probenecid with benzyl protected glucose.

The Yu group reported a method to synthesize *β*-glycosyl esters, by employing 1-hydroxybenzotriazole (HOBt) as the acyl transfer reagent and DABCO as the base [37]. When 2,3,4,6-tetra-*O*-benzyl-D-glucopyranose **11** was condensed with benzoic acid **65** in the presence of HOBt, EDCI, and DABCO in CH_3_CN at room temperature, it selectively afforded *β*-glycosyl esters **66** in excellent yield (Scheme 23a). Aromatic carboxylic acids with electron-withdrawing groups and electron-donating groups at various positions were tolerated and delivered the desired *β*-glycosyl esters in moderate-to-excellent yields and with almost complete stereo control (Scheme 23a). It is noteworthy that the heteroaromatic carboxylic acids were also well tolerated, as **67** was successfully coupled with hemiacetal with excellent stereoselectivity (Scheme 23b). Aliphatic carboxylic acids were also competent substrates. Carboxylic acid-containing drugs, for example Probenecid **69**, were smoothly linked to the glucosyl units (Scheme 23c). However, salicylic acid and furosemide failed to be transformed to the corresponding glucose conjugates, possibly due to the existence of acidic phenol and sulfonamide groups. Different sugars like pyranosyl hemiacetals derived from D-glucose, D-galactose, 2-deoxy-D-glucose, 2,3-dideoxy-D-glucose, D-xylose, and D-mannose were successfully acylated with good yields and excellent stereoselectivities. However, despite the satisfactory yields, furanosyl hemiacetals showed almost no stereoselectivity. The loss of stereo control could be due to the steric environments of anomeric hydroxyls in the furanoses. According to the mechanistic studies, stereoselective synthesis of glycosyl ester involved dynamic kinetic esterification, in which the more reactive *β*-anomer was preferred.

**Scheme 24:**
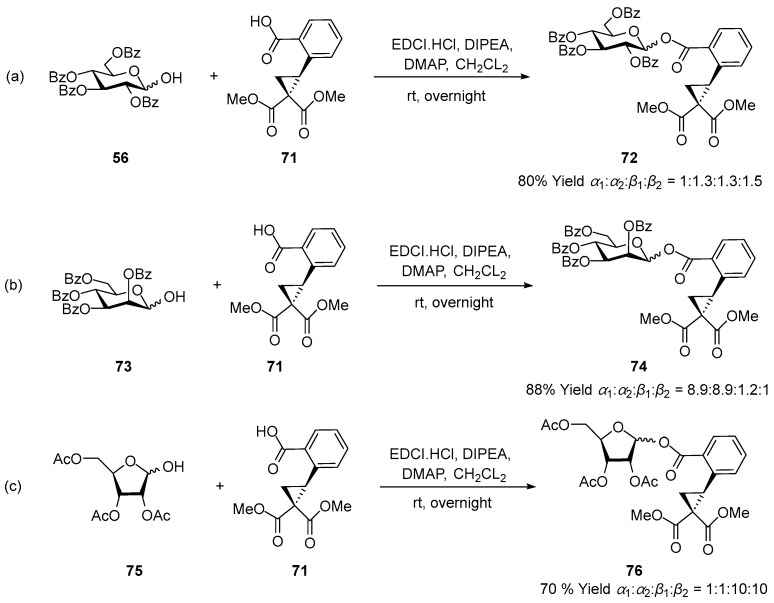
(**a**) Synthesis of glucosyl CCBzs Donors. (**b**) Synthesis of mannosyl CCBzs Donors. (**c**) Synthesis of ribofuranosyl CCBzs Donors.

Compounds with ring strain, such as donor–acceptor cyclopropanes (DACs) and donor–acceptor cyclobutanes (DABs) [38,39], possess high π-character and readily undergo homologous conjugate addition with various nucleophiles in either intermolecular or intramolecular fashion, initiated by a non-covalent chelation process. Catalytic glycosylation reactions using cyclopropane-fused glycosyl donors have achieved remarkable control over anomeric selectivity, enabling the synthesis of unnatural glycosides [40]. This approach provides an efficient method for accessing natural sugar derivatives through catalytic, strain-release-driven glycosylation [41]. In 2023, Liu and coworkers reported a method to synthesize glycosyl donor with an intramolecularly incorporated DAC [42]. They designed a glycosyl *ortho*-2,2-dicarbonylcyclopropylbenzoate, by the coupling reaction between anomeric hemiacetals **56** and *ortho*-2,2-dimethoxycarbonylcyclopropylbenzoic acid (CCBzOH) **71** in the presence of EDCI, DIPEA, and DMAP to afford glycosyl CCBzs **72** in good-to-excellent yields. Different sugar molecules (glucose, galactose, mannose, ribofuranose, etc.) with different protecting groups (benzoyl, acetyl) were successfully applied to this methodology (Scheme 24).

These glycosyl donors were used for glycosylation by the activation of Lewis acid, which selectively chelates with two carbonyl groups to activate cyclopropyl group. The nucleophilic attack of the carbonyl group in the benzoyl group opens the cyclopropyl group to form the enolate, accompanied by the cleavage of the anomeric C-O bond to generate the oxocarbenium, which is then attacked by an incoming acceptor nucleophile, giving the desired products. The enolate produced not only acts as the leaving group but also captures the free proton released by the acceptor to maintain the reaction system in a nearly neutral state.

**Scheme 25:**
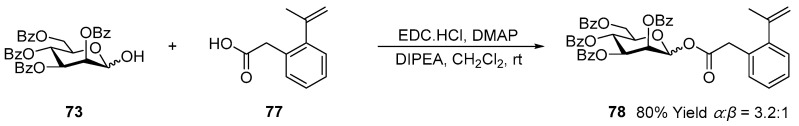
Synthesis of GIPPAS.

Li group reported the synthesis of disarmed glycosyl *ortho*-isopropenylphenylacetates (GIPPAs) by reacting protected hemiacetals **73** with *ortho*-isopropenylphenylacetic acid **77 [43]**. Using (1-(3-dimethylaminopropyl)-3-ethyl-carbodiimide hydrochloride) (EDC.HCl) as the coupling reagent in the presence of DIPEA and catalytical amounts of DMAP, the reaction afforded GIPPAs **78** in good yield (Scheme 25). Different sugar hemiacetals, like gluco-, galacto-, manno-, xylo-, and rhamnopyranosyl, were applied to this method with varying stereoselectivity. D-mannosyl, L-rhamnosyl, and ribofuranosyl IPPAs were isolated with 1,2-trans anomers as the major yields, whereas 2-deoxy-2-phthylimido-D-glucosyl primarily gave *β*-anomer. The formation of other GIPPAS was less selective and gave a mixture of anomers.

**Scheme 26:**
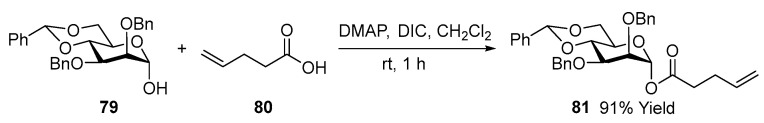
Synthesis of mannosyl pentenoate.

Kim and coworkers synthesized Mannosyl pentenoate donors in the presence of *N*, *N*-diisopropylcarbodiimide (DIC) coupling reagent [44]. The treatment of *α*-anomer of protected mannopyranose **79** with 4-pentenoic acid **80** in the presence of a catalytic amount of DMAP and DIC reagent afforded 4,6-*O*-benzylidene mannosyl pentenoate **81**, which retained an anomeric configuration, in excellent yield (Scheme 26). The reaction mechanism here involved the formation of an isourea type of intermediate through the reaction of carboxylic acid with DIC reagent. The nucleophilic oxide generated due to the deprotonation of anomeric OH under basic conditions attacked the isourea type of intermediate and expelled a urea byproduct to afford ester **81** as the desired glycosyl donor.

**Scheme 27:**
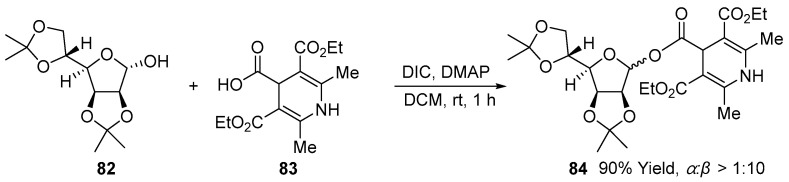
Esterification of hemiacetal assisted by DIC.

Diao and coworkers reported a method to synthesize C-aryl and heteroaryl glycosides from glycosyl esters [45]. The glycosyl esters used in this methodology were synthesized by coupling of protected furanose or pyranose sugars with dihydropyridine (DHP) carboxylic acid derivatives. When diacetonide-*α*-D-mannofuranose **82** was treated with DHP carboxylic acid derivative **83** in the presence of *N*,*N*-diisopropylcarbodiimide (DIC) or EDCI reagent, and a catalytical amount of DMAP, and *O*-mannofuranosyl ester was obtained in excellent yield with a predominant *β*-anomer (Scheme 27). Different sugar substrates like ribose, glucose, galactose, and arabinose were all applicable.

**Scheme 28:**
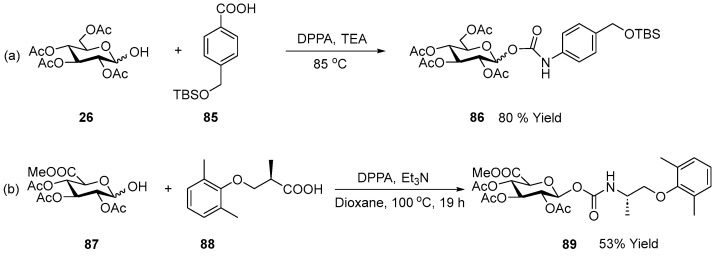
(**a**) Synthesis of glycosyl carbamates using benzyl acid derivative. (**b**) Synthesis of glycosyl carbamate using propanoic acid derivative.

During the synthesis of an amphiphilic prodrug, Wang et al. coupled the sugar moiety with a benzyl acid derivative under basic conditions [46]. The treatment of acetyl-protected hemiacetal **26** with para substituted benzyl acid derivative **85** in the presence of diphenylphosphoryl azide (DPPA) and triethylamine (TEA) afforded glycosides **86** with a carbamate linkage (Scheme 28a). The reaction was initiated with the activation of carboxylic acid by DPPA to form an azide-containing intermediate, which underwent Curtius-type rearrangement to generate an isocyanate intermediate. The nucleophilic attack from anomeric OH of the isocyanate formed a carbamate linkage.

The same methodology was adopted by Lentini and coworkers in the synthesis of *N*-carbonyloxy *β*-D-glucuronide of mexiletine (sodium salt) [47]. When acetyl-protected D-glucopyranose **87** was treated with the prepared carboxylic acid synthon **88** in the presence of DPPA and triethylamine, the corresponding carbamate **89** was afforded as *β-*anomer (Scheme 28b). The *β-*selectivity is presumably due to the higher nucleophilicity of the *β*-1-OH group than that of the *α*-anomer.

**Scheme 29:**
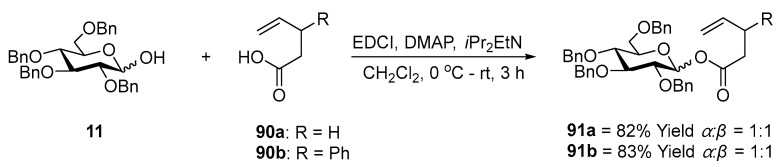
Synthesis of pentenoate glycosyl donors.

The Mandal group reported a method to synthesize substituted pentenoate donors under basic conditions [48]. The treatment of glycosyl hemiacetals **11** with phenyl-4-pentenoic acid or 4-pentenoic acid **90** in the presence of EDCI, DMAP, and DIPEA under Steglich esterification conditions afforded the desired glycosyl donors **91** in good yields with low selectivity (Scheme 29). Different sugar substrates like gluco-, galacto-, manno-, and rhamnopyranosyl were all tolerated.

Wang et al. reported the synthesis the amino acid glucose ester by the treatment of benzyl-protected glucose with L-lysine and L-glutamic acid using EDCI as a coupling reagent in the presence of DMAP, which afforded the desired ester as a mixture of anomers with low selectivity in moderate yield [49].

**Scheme 30:**
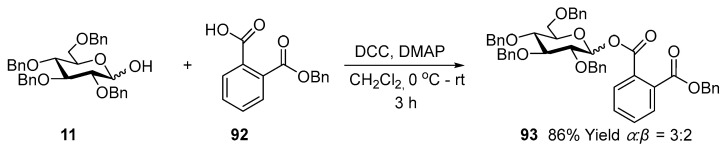
Synthesis of benzyl phthalate glycosyl donors.

The Kim group synthesized glycosyl donors with benzyl phthalate as the leaving group [50]. Esterification of *O*-benzyl-D-glucose **11** with benzyl hydrogen phthalate **92** using DCC in the presence of a catalytical amount of DMAP yielded the glucopyranosyl benzyl phthalate donors **93** as a mixture of anomers with low selectivity (Scheme 30). Various sugars like benzyl- and acetyl-protected mannopyranose and 2-deoxyglucopyranose were also applied to the method.

**Scheme 31:**
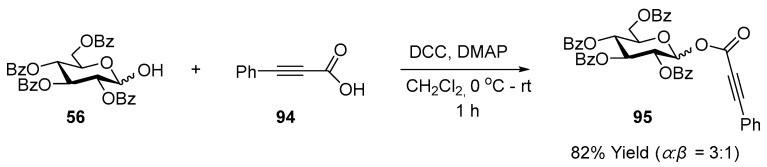
Synthesis of phenylpropiolate glycosyl (PPG) donors.

Amit Kumar and colleagues reported a method to synthesize phenylpropiolate glycosyl (PPG) donors [51]. Coupling of hemiacetals **56** with phenylpropiolic acid **94** in the presence DCC and DMAP afforded propiolate-based glycosyl donors **95** as a mixture of anomers with high *α*-selectivity (Scheme 31). Different types of carboxylic acids, for instance, propiolic acid, 2-hexynoic acid, and 2-butynoic acid, were also used as substrates, which afforded the desired glycosyl donors as mixtures of anomers.

**Scheme 32:**
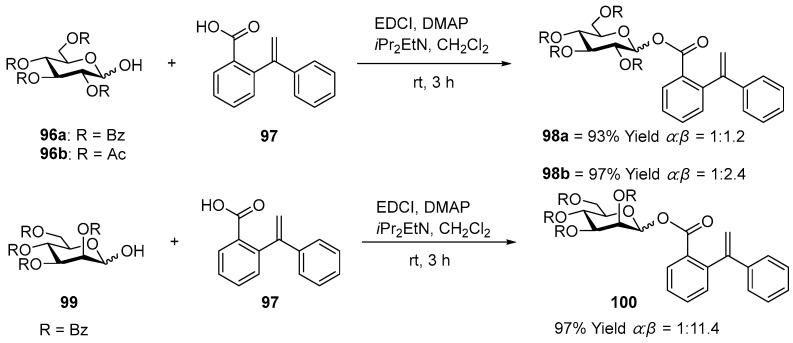
Formation of PVB glycosyl donors.

The Xiao group reported the synthesis of another type of ester donor *o*-(1-phenylvinyl)benzoate (PVB) [52]. The condensation of sugar hemiacetals **96** with *o*-(1-phenylvinyl)benzoic acid **97** in the presence of 4-dimethylaminopyridine (DMAP), *N*-(3-dimethylaminopropyl)-*N*-ethylcarbodiimide hydrochloride (EDCI), and diisopropylethylamine (DIPEA) afforded PVB donors **98** in excellent yields with *β*-anomer as the major product (Scheme 32). Several sugars like gluco-, galacto-, manno-, rhamno-, and xylopyranose with acetyl- and benzoyl-protecting groups were all successfully applied to the methodology.

**Scheme 33:**
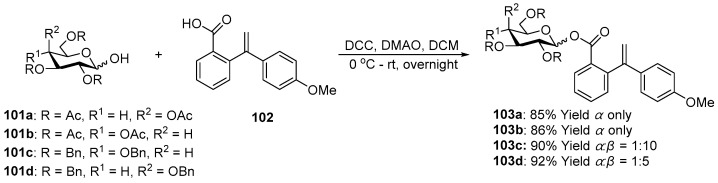
Formation of PMPVB glycosyl donors.

The Kancharla group also synthesized (*p*-MeO)phenylvinylbenzoate (PMPVB) donors, which were more reactive than PVB donors [53]. In this methodology, hemiacetals **101** were coupled with *O*-[1-(*p*-methoxyphenyl)vinyl] benzoic acid **102** by using DCC as a coupling reagent in the presence of DMAP. Interestingly, the armed and disarmed donors were yielded with opposite stereoselectivities (Scheme 33). Glucose- and galactose-derived disarmed donors **103a**, **103b**, along with the 6-OTBDPS-protected triacetyl glucose-derived donor, were obtained as *α*-isomers only, whereas the 6-OTBDPS-protected triacetyl galactose donor and benzoyl-protected galactose donor were formed as a mixture of anomers with high *α*-selectivity. In contrast, the armed benzyl- **103c**, **103d** and *p*-Me-benzyl-protected donors were obtained as a mixture of anomers with high *β*-selectivity.

### 2.5. Mitsunobu Reaction and Appel Reaction

The Mitsunobu reaction is well known for converting a hydroxyl group into esters or phenyl ethers with inverted stereochemistry at the carbon center under mild conditions. It has been applied to carbohydrate chemistry in the modification of anomeric hydroxyl to esters or phenyl glycosides [54]. Theoretically, if pure anomers are used, it should exclusively provide the glycoside with the opposite anomeric configuration. A few newly published studies are discussed to summarize the recent progress in this area. The most noteworthy observation of the Mitsunobu reaction in anomeric conversion is that the extraordinary stereoselectivity of this reaction hypothetically claimed by the S*_N_*2 mechanism to give stereo-inverting products is challenged. When sterically hindered, sugar hemiacetal is subject to the Mitsunobu conditions, and the hypothesized intermediate, the glycosyloxyphosphonium ion, is less stable and decomposes into the corresponding oxocarbenium ion, which would then react with nucleophiles in an S*_N_*1 mechanism to generate a mixture of anomers.

**Scheme 34:**
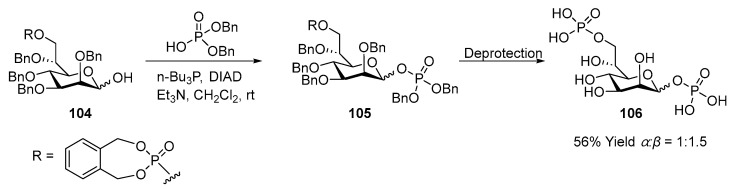
Formation of Heptose bisphosphate via Mitsunobu reaction.

The Inuki group synthesized D-glycero-D-manno-Heptose 1,7-bisphosphate (HBP), the precursor for heptose residues found in Gram-negative bacterial membrane surface glycoproteins and glycolipids, by the Mitsunobu reaction [55]. The treatment of mannose-6-phosphate derivative **104** with dibenzyl phosphate under Mitsunobu conditions in the presence of triethyl amine afforded the desired bisphosphate **106** as anomeric mixtures (Scheme 34). The low selectivity is proposed to be derived from the formation of an oxocarbenium species leading to an S*_N_*1-type reaction.

**Scheme 35:**
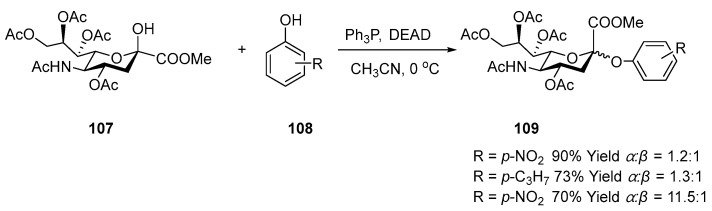
Synthesis of aryl sialosides via Mitsunobu reaction.

Gao et al. used the Mitsunobu reaction for the efficient synthesis of aryl *α*/*β*-sialosides [56]. The treatment of salic acid derivative **107** with different phenol derivatives **108** in the presence of Ph_3_P and diethyl azodicarboxylate (DEAD) in acetonitrile resulted in the formation of sialosides **109** with moderate anomeric selectivity (Scheme 35). The yields of the products are in accordance with the acidity of the phenol. Phenols bearing electron-withdrawing substituents gave excellent yields, while phenols substituted with electron-donating groups resulted in lower yields and increased amounts of side products. However, no correlation was observed between the pK_a_ of the phenols and the stereoselectivity. Again, the comptonization of stereoselectivity was ascribed to the formation of oxocarbenium ions and the S*_N_*1-type reaction mechanism.

In 2020, Somsak and coworkers obtained *α-*sialosides from a salic acid derivative by the Mitsunobu reaction [57]. The treatment of the salic acid derivative with phenol derivative **108** in CH_3_CN, and by dropwise addition of DEAD at −30 °C, afforded corresponding sialosides **109** with predominant *α*-anomer (Scheme 35). A trace amount of byproduct was also seen. In this method, there was significant improvement in stereoselectivity as compared to Gao’s methodology. It was observed that lowering the temperature was beneficial for *α*-selectivity of these reactions.

**Scheme 36:**
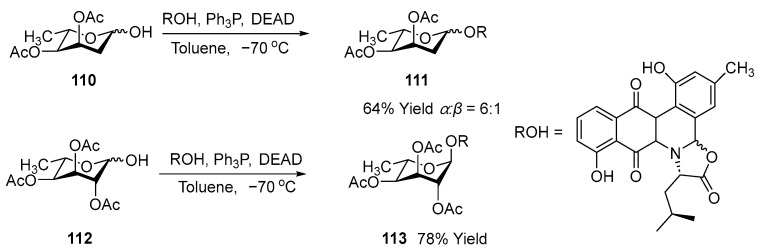
Synthesis of jadomycins enabled by Mitsunobu reaction.

The Yu group synthesized various jadomycins using sugars devoid of a C-2 participating group by the Mitsunobu reaction [58]. When **110** was treated with phenolic aglycon, it afforded glycoside **111** with predominantly *α-*configuration, while sugar containing the C-2 participating group resulted in the formation of exclusively 1,2-*trans*-glycoside **113** (Scheme 36). The involvement of the neighboring group participation could indicate an S*_N_*1-type reaction mechanism.

**Scheme 37:**
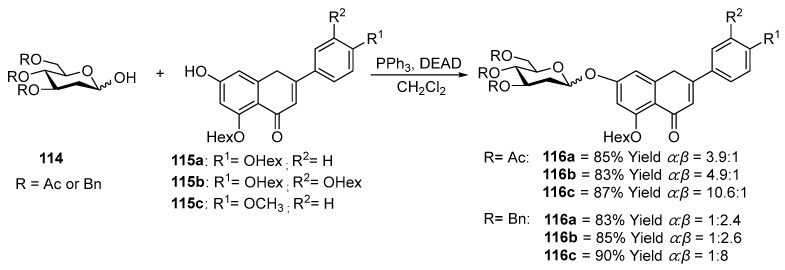
Mitsunobu reaction of deoxy sugars with different flavonoids.

Gao and coworkers reported a method for the stereoselective synthesis of flavonoid 2-deoxyglucosides using the Mitsunobu reaction [59]. The treatment of 3,4,6-tri-*O*-acetyl-2-deoxylgucose **114** with 7-hydroxyl apigenin **115** afforded flavonoid 2-deoxyglucosides **117** in good yield and gave *α*-selectivity (Scheme 37). However, when 7-hydroxyl flavonoids were reacted with 3,4,6-tri-*O*-benzyl-2-deoxy-D-glucopyranose, the obtained glucosides had predominant *β*-configuration (Scheme 37). The reaction appeared to be S*_N_*2-like with the displacement of an oxyphosphonium salt intermediate since the observed *β*:*α* stereoselectivity is in accordance with the *α*:*β* anomeric composition of the 2-deoxyglucose starting material. The protecting groups played a crucial role by controlling the *α*:*β* anomer ratio of the 2-deoxysugar. When the 2-deoxysugar was benzylated, termed “armed”, the substrate had a greater *α*-anomeric preference because of the electron-donating benzyl groups, resulting in the formation of *α-*configured oxyphosphonium intermediate, and therefore *β*-selectivity. In contrast, the “disarmed” electron-withdrawing acetylated substrate had much less *α*-anomeric stabilization, resulting in more *β*-configured oxyphosphonium intermediate, giving selectivity for *α*-glycosides. Additionally, the nucleophilicity of different flavonoid 7-*O*-phenoxides also affected the stereoselectivities.

**Scheme 38:**
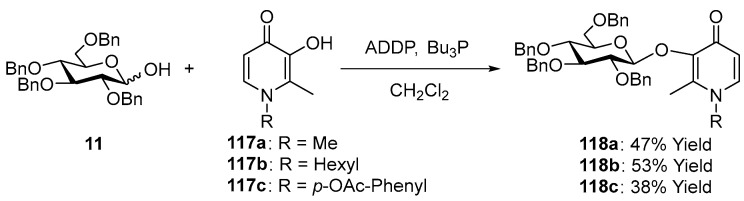
Synthesis of pyridionone glycosides using Mitsunobu reaction.

The Orvig group reported a method to synthesize pyridionone glycosides using the Mitsunobu reaction [60]. The treatment of 2,3,4,6-tetra-*O*-benzyl-D-glucopyranose **11** with 3-hydroxy-4-pyridinones derivatives **117** in the presence of 1,1′-(Azodicarbonyl)dipiperidine (ADDP) and Bu_3_N afforded pyridionone glycosides **118** with *β*-selectivity in moderate yields (Scheme 38). The *β*-selectivity was ascribed to the high percentage of *α*-anomer (80–90%) in the starting material that underwent an S*_N_*2-type reaction to yield an inverted anomeric configuration.

### 2.6. Cross-Coupling

Transition metal-catalyzed cross-coupling reactions are useful tools for C-O bond formation and have been applied to carbohydrate chemistry. Like the coupling by carbodiimide and the Mitsunobu reaction, cross-coupling reactions are also mild reactions with exceptional substrate tolerance. In terms of atom economy, they are better than the other two reactions, which require an excess of reagents in addition to reactants, since only catalytical amounts of transition metals are needed. The substrate scope is limited since it is difficult to couple *sp*^3^ carbon with anomeric oxygen.

**Scheme 39:**
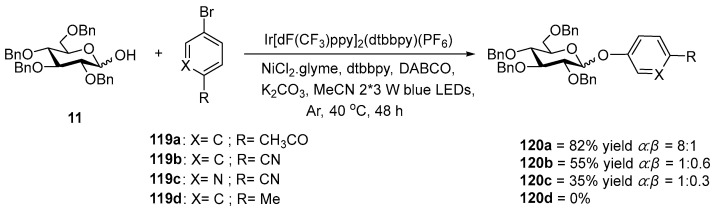
Dual nickel/photoredox catalyzed cross-coupling of glycosyl hemiacetals with aryl bromides.

In 2015, the MacMillan group developed a methodology to generate C-O bonds using electron-deficient aryl bromides and alcohols by synergistically combining photoredox and metal catalysis [61], a method originally reported by Osawa et al.[62]. The methodology was then used by the Xiao group to synthesize *O*-aryl glycosides. From glycosyl hemiacetals, they synthesized phenyl glycosides using dual photoredox and nickel-catalyzed cross-coupling with aryl bromides under mild conditions [63]. The reaction of ether-protected carbohydrate hemiacetals **11** with aryl bromides **119** in the presence of Ir[dF(CF_3_)ppy]_2_(dtbbpy)PF_6_ and NiCl_2_·glyme as photocatalysts under basic conditions afforded O-aryl glycoside **120** in low-to-excellent yields (Scheme 39). Generally, the glycosides produced were either exclusively or extensively *α-*anomers. It was assumed that the extended reaction time (48 h) amplified the *α*-selectivity caused by the anomeric effect on the *α*-major hemiacetal.

Pyranose and furanose sugars, and even disaccharides, were well tolerated. However, acetyl-protected donors were inert to these reaction conditions, presumably due to the electron-withdrawing property of ester groups. Several different electron-deficient aryls (**119a**, **119b**) and heteroaryl bromides (**119c**) were explored as coupling partners and generated *O*-aryl glycosides in low-to-moderate yields. Aryl bromides with electron-donating alkyl substituents (**119d**) did not react. Although limited in substrate scope, this method is a useful addition to the current synthetic arsenal for the synthesis of phenolic glycosides, which have broad application in carbohydrate chemistry.

**Scheme 40:**
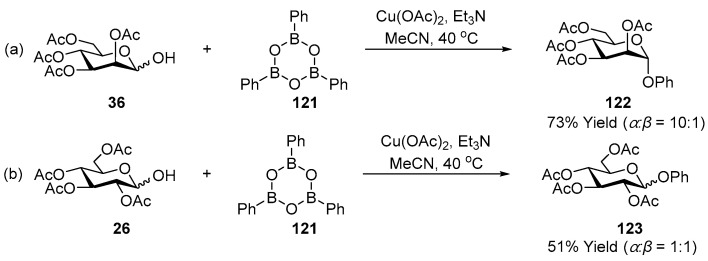
(**a**) Copper-catalyzed cross-coupling of anomeric OH group of mannose with triarylboroxine. (**b**) Copper-catalyzed cross-coupling of anomeric OH group glucose with triarylboroxine.

Encouraged by ChamLam-Evans cross coupling of 1-aminosugars with arylboronic acids under copper catalysis, many research groups have tried to form *O*-glycosides using their strategy [64]. Taylor and coworkers were the first to report the synthesis of *O*-glycoside from carbohydrate hemiacetals and arylboronic acids [65]. They showed that *O*-arylation of sugar hemiacetals could be achieved efficiently at room temperature in air with a stoichiometric amount of Cu(OAc)_2_. When protected hemiacetal sugar **36** was treated with a stoichiometric amount of Cu(OAc)_2_, triethylamine, and a triarylboroxine **121** (prepared by dehydration of corresponding arylboronic acid) in acetonitrile under ambient conditions, *O*-aryl glycosides **122** was produced. Gluco-, galacto-, and manno-configured pyranoses with benzyl, acetyl, benzylidene, and isopropylide protecting groups were all tolerated. *O*-phenyl mannofuranosides and pyranoside derivatives were obtained with high *α*-selectivity (Scheme 40a) but the reaction of gluco/glucopyranose and arabinofuranose substrates generated mixtures of anomers (Scheme 40b). The anomeric mixtures were likely to arise from competing steric and electronic factors, as well as relative acidities. The thermodynamically more stable *α*-anomer is also less sterically hindered and reacts faster to predominantly give an *α* product.

**Scheme 41:**
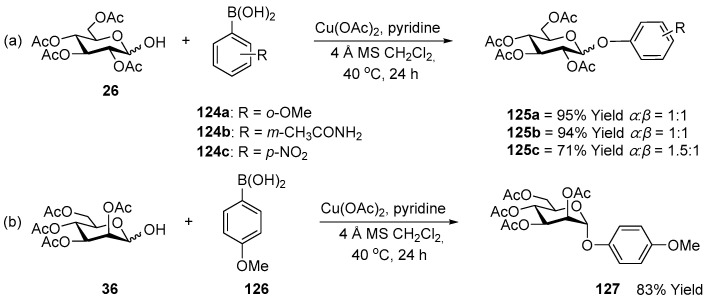
(**a**) Cross-coupling of glycosyl hemiacetals with aryl boronic ester enabled by copper catalysis. (**b**) Synthesis of phenolic glycosides of mannose by copper-catalyzed cross coupling reaction.

In 2019, Ye and coworkers reported an efficient method for the synthesis of phenolic glycosides by copper-mediated coupling reactions [66]. When an *O*-protected lactol **26** was treated with aryl boronic acid **124** bearing either electron-donating or electron-withdrawing substituents in the presence of a stoichiometric amount of Cu(OAc)_2_, pyridine, and molecular sieves in DCE at 40 °C, the reaction afforded the phenolic glycosides **125** in moderate-to-excellent yields (Scheme 41). Gluco-, galacto-, and manno-configured hemiacetals successfully underwent cross-coupling. Most of the reactions afforded anomeric mixtures except mannose-derived lactols, which provided phenolic glycosides with *α-*configuration (Scheme 41b). Electron-withdrawing and electron-donating substituents at *ortho*, *meta*, *para* positions on arylboronic acids all generated the *O*-glycosides in high yields (Scheme 41a).

**Scheme 42:**
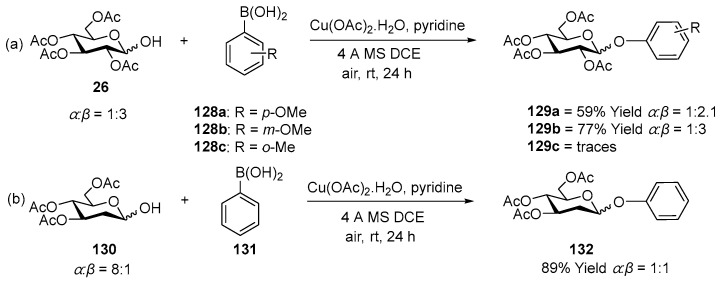
(**a**) Copper-catalyzed cross-coupling of glycosyl hemiacetal with arylboronic acids. (**b**) Cross coupling of 2-deoxy glucopyranoside with arylboronic acids.

As a continuation of work on C-O bond formation, Messaoudi and coworkers reported a method in which hemiacetals can be efficiently *O*-arylated using a catalytic amount of Cu(OAc)_2_ at room temperature (rt) under an air atmosphere [67]. When *O*-acetal-D-glucopyranose **26** was treated with various arylboronic acids **128** in the presence of Cu(OAc)_2_, pyridine, and molecular sieves, it afforded aryl *O*-glycosides as a mixture of *α-* and *β-*anomers **129** (Scheme 42a). The use of molecular sieves in the reaction played an important role as it not only improved reaction efficiency, but also influenced the *α*:*β* ratios of the *O*-aryl glycosides produced. In the presence of molecular sieves, the *α/β* ratio was close to the composition of the hemiacetal anomers (Scheme 42a), while in their absence, the starting material underwent anomerization. An exception is the reaction between phenylboronic acid and 2-deoxy glucopyranoside, which afforded 1:1 mixtures of *O*-aryl glycoside even in the presence of molecular sieves. These results indicated that the acetyl group at the C2 position is crucial for preserving the anomeric purity of glycosyl lactols (Scheme 42b). Electron-rich and electron-poor boronic acids were both coupled with hemiacetals with comparable efficiencies. Compared to *meta*- and *para*-substituted boronic acids, *ortho*-substituted derivatives are less reactive and only produced trace amounts of the desired *O*-aryl glycosides (Scheme 42a). These cross-coupling reactions demonstrated good functional group tolerance and gave stereo-retentive *O*-aryl glycoside under mild conditions. The stereo outcome is normally dictated by the configurational composition of the hemiacetal reactants, which may allow further exploration.

**Scheme 43:**
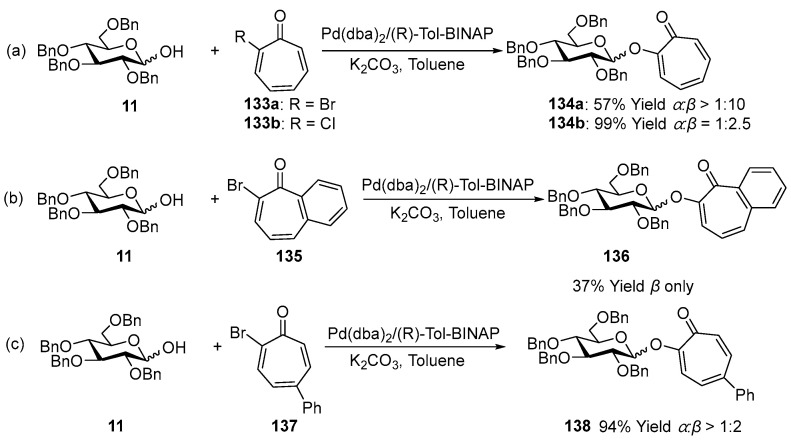
(**a**) Synthesis of tropolone glycosides using 2-bromotropone and 2-chlorotropone. (**b**) Synthesis of tropolone glycosides using benzannulated troponoid. (**c**) Synthesis of tropolone glycoside using aryl substituted 2-bromotropone.

The Banwell group reported a method to synthesize tropolone glycosides by Pd-catalysis [68]. The treatment of protected glucose **11** with 2-bromotropone **133a** in the presence of a Pd catalyst and K_2_CO_3_ afforded the desired glycosides **134a** with predominant *β*-selectivity in excellent yield (Scheme 43a). Different sugar substrates like galactose, xylose, mannose, and lactose were well tolerated. D-mannose and L-rhamnose tropolone glycosides were isolated as *α*-anomers. The nature of ligands used influenced the ratios of the anomers in the reaction. When XantPhos were employed as the ligand, the *α*-anomer was the dominant product while (R)-Tol-BINAP preferably gave *β*-anomers. The substrate scope of troponoid was explored by reacting with a glucose derivative. Troponoid containing an electron-rich substituent resulted in no reactions. A chloro analogue of bromotropone **133b** was proven to be a competent substrate for coupling. Benzannulated troponoid **137**5 gave modest yields of the products (Scheme 43b), while aryl-substituted substrates **137** provided excellent yields (Scheme 43c).

### 2.7. Nucleophilic Aromatic Substitution (S_N_Ar)

S*_N_*Ar reactions are between strong nucleophiles, such as hydroxide, and aryl compounds with strong electron-withdrawing groups at the para position. In basic conditions, the anomeric hydroxyl group of sugars can be deprotonated and converted to hydroxide, which can serve as nucleophiles for S*_N_*Ar reactions to produce aryl glycosides. Heterocyclic compounds are widely used in medicinal chemistry because of their versatility in tuning the polarity, hydrophilicity, and H-bonding ability of drug candidates. Therefore, the installation of heteroaryl on carbohydrates is of special interest.

**Scheme 44:**
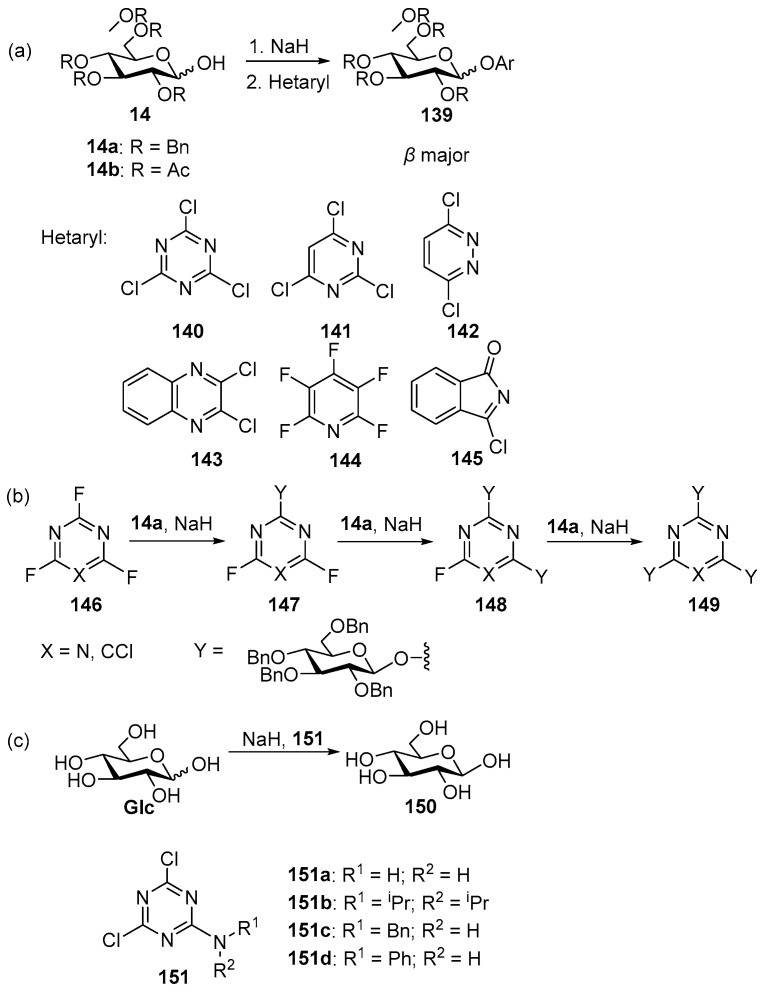
(**a**) Anomeric *O*-hetarylation of carbohydrate hemiacetal via S*_N_*Ar reaction. (**b**) Synthesis of mono/di/tri-glycosylated triazine by consecutive hetarylation. (**c**) Anomeric *O*-hetarylation of unprotected hemiacetal.

In an effort to search for new leaving groups in glycosylation, Schmidt and coworkers explored a series of electron-deficient heteroaryls reacting with carbohydrates by the S*_N_*Ar mechanism [69]. Firstly, the anomeric hydroxyl group of 2,3,4,6-tetra-O-benzyl-D-glucose or 2,3,4,6-tetra-O-acetyl-D-glucose was deprotonated by treatment with NaH in various solvents. The resulting hydroxides were subject to hetarylation and successfully afforded hetaryl glucopyranosides (Scheme 44a). The higher reactivity of equatorial oxide over axial oxide caused by the anomeric effect dictated the stereoselectivity and produced glycosides with primarily *β*-configuration. Hetaryls with halide, including cyanuric chloride derivatives **140**–**143**, pentafluoropyridine **144**, and imide halides **145**, are all compatible with the reaction condition. When more reactive cyanuric fluoride is used, consecutive S*_N_*Ar could happen to produce mono/di/tri-glycosylated triazine **147**–**149** (Scheme 44b). The anomeric acetal hydroxyl group can be selectively deprotonated over alkyl hydroxyl due to higher acidity. As shown in Scheme 1c, when unprotected glucose was treated with sodium methoxide followed by 2,4-dichloro-1,3,5-triazine derivatives **151**, mono-substituted *β*-products were acquired in good yields. Following these studies, the researchers investigated the glycosylation reaction with these hetaryl glycosides as donor. Although only moderate yields of disaccharides were obtained with unsatisfactory stereoselectivity, this research pioneered the screening of heterocycle leaving groups in glycosylation and the systematic study provided insights for future design of aryl nucleofugal leaving groups.

Glycosyl phenols carrying an electron-withdrawing substituent, such as the nitro group, were synthesized and tested as donors in glycosylation, but were found to be not reactive enough to produce nucleophiles in reaction media [70]. To obtain more reactive aryl glycosyl donors, Jensen et al. reported a method by S*_N_*Ar reactions [71]. Using a double base system of an organic base and a catalytic amount of inorganic base (Li_2_CO_3_), they synthesized glycosides of methyl 2-hydroxy-3,5-dinitrobenzoate (DISAL) and methyl 4-hydroxy-3,5-dinitrobenzoate.

**Scheme 45:**
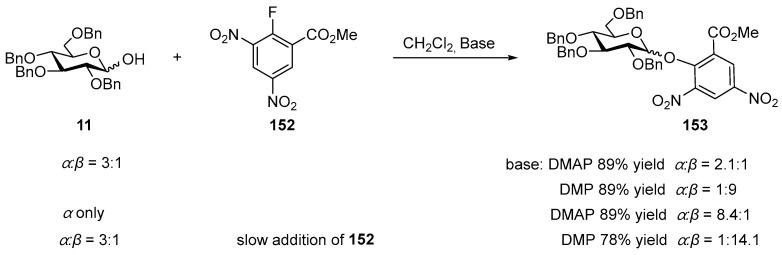
Synthesis of DISAL donors.

When benzoyl-protected glycopyranose **11** and methyl ester **152** were reacted in the presence of 4-(*N*, *N*-dimethylamino) pyridine (DMAP) (Scheme 45), the reaction condition produced an *α*/*β* ratio comparable to that of the 1-OH derivative. Interestingly, when DMAP was replaced with 1,4-dimethylpiperazine (DMP) (Scheme 45), the stereoselectivity was inverted and *β*-glycoside was obtained as the major product, although longer reaction times were needed. A thorough screening of possible soluble bases was conducted and only DMP and DMAP were effective.

The *α*- and *β*-anomer could be synthesized selectively. When twice-recrystallized tera-*O*-benzyl glucose was used under DMAP conditions, *α*-anomer was obtained with a small amount of *β*-anomer. This indicated that even in quick reactions, minor anomerization of 1-OH happens before the formation of aryl glycoside. To selectively make only *β*-glycoside, a fluorobenzene derivative was slowly added to a sugar and DMP solution to selectively react with the more reactive *β-*anomeric oxide. When the *β*-glycoside was separated, a small amount of α-anomer was also recovered. It should also be noted that DISAL donors do not undergo anomerization in non-polar conditions used for their synthesis. After synthesis of DISAL donors, their potential as glycosyl donors was demonstrated by reacting with simple alcohols like methanol to stereoselectively give glycosylated products.

**Scheme 46:**
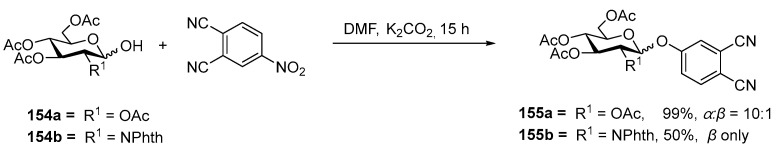
Synthesis of cyanophenyl glycosides.

Ziegler and colleagues reported a method for the synthesis of phenyl glycosides by the displacement of the nitrite group on substituted nitrobenzene with anomeric OH of hemiacetals via the S*_N_*Ar mechanism [72]. The treatment of protected hexopyranoses **154a** with nitro- and cyano-substituted benzenes in the presence of NaH or K_2_CO_3_ in DMF yielded the corresponding glycoside **155a** with thermodynamically controlled *α*-selectivity. The presence of the phthalimido group at the C2 position in **154b** resulted in *β*-anomer **155b** (Scheme 46). Sugar substrates with thionucleophiles were also applicable for the displacement of nitrite groups through the S*_N_*Ar mechanism.

**Scheme 47:**
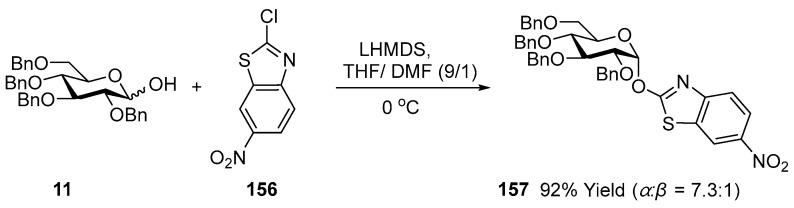
Synthesis of glycosyl benzothiazoate donor.

The Mukaiyama group continued their journey to synthesize a characteristically similar glycosyl donor which contained 6-nitro-2-benzothiazoate as the leaving group [73]. The direct condensation reaction between anomeric hydroxyl group of 2,3,4,6-tetra-*O*-benzyl- D-glucopyranoside **11** and 2-chloro-6-nitrobenzothiazole **156** in the presence of Lithium bis(trimethylsilyl)amide (LHMDS) provided glycosyl benzothiazoate **157** with *α* and *β* mixtures (Scheme 47). Glycosyl donors that contained non-substituted benzothiazole were unstable and were partially hydrolyzed during the work-up process. The presence of an electron-withdrawing nitro group contributed to the stability of the formed glycosyl donor.

### 2.8. Arylation of Anomeric Hydroxyl via Elimination–Addition (EA)

The elimination–addition reaction that takes place on aromatic compounds can also be used to produce aryl glycosides. The mechanism involves the elimination of the leaving group from the aromatic compound under basic conditions and the generation of aryne to be attacked by glycosyl oxide. Arynes have been used for the *O*-arylation of alcohols and aromatic compounds because of their ring strain and strong electrophilicity. *O*-arylation of arynes only requires a strong base, and a simple reaction procedure can be advantageous.

**Scheme 48:**
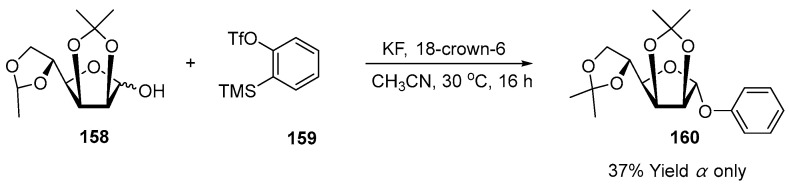
Preparation of aryl ethers via aryne intermediates.

Mukherjee and coworkers used silyl aryl triflates, which can generate benzyne intermediates in mild basic conditions, for O-arylation of carbohydrates by the elimination–addition mechanism to generate medicinally important O-aryl glycosides [74]. When a protected hemiacetal, 2,3,5,6-bis-isopropylidene mannofuranose **158**, was treated with silyl aryl triflate **159** under basic conditions, the desired *O*-arylated glycosides of mannose **160** were obtained in low yields (Scheme 48). According to the mechanistic studies, benzyne is produced by fluoride-induced breakdown of 2-(trimethylsilyl) phenyl trifluoromethanesulfonate **159** and underwent an addition reaction with the OH group of **158** to produce aryl products. Several reactions involving carbohydrate-derived diols showed that primary over secondary and equatorial over axial OH groups are preferred, consistent with the order of nucleophilicity.

### 2.9. Miscellaneous

#### 2.9.1. Reaction of Anomeric Hydroxyl with Cationic Species

**Scheme 49:**
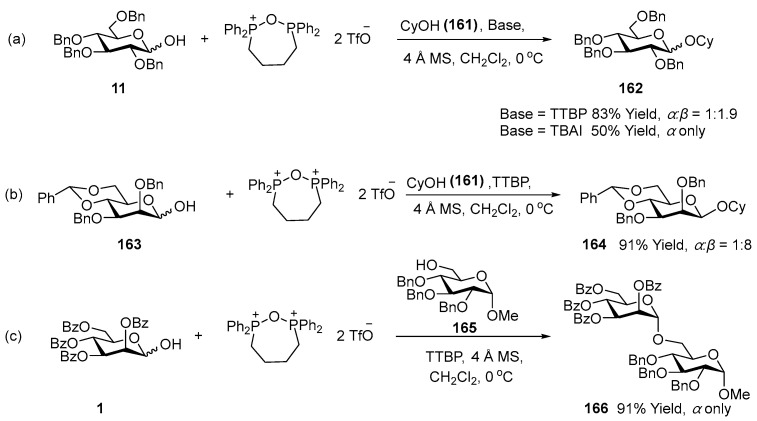
(**a**)Synthesis of glycosides by dehydrative glycosylation. (**b**) Dehydrative glycosylation of benzylidene protected mannose substrate. (**c**) Dehydrative glycosylation of benzoyl protected mannose substrate.

The Walczak group reported a method for dehydrative glycosylation of C1-hemiacetals by using bis-phosphine oxides and triflic anhydride as coupling reagents [75]. These reagents were chosen for their strong electron-withdrawing effects to enhance the electrophilicity of the adjacent carbon center. Different types of cyclic phosphonium anhydrides were prepared by reacting bis-phosphine oxides with Tf_2_O. The treatment of benzyl-protected glucopyranose **11** with cyclic phosphonium anhydride in the presence of TTBP base followed by the addition of cyclohexyl alcohol **161** resulted in the formation of glycoside **162** with high *β*-selectivity. When TTBP was replaced by tetra-*n*-butylammonium iodide (TBAI), only *α*-glycoside was isolated, with decreased yield (Scheme 49a). Different sugar substrates such as deoxysugars and ribofuranose were easily glycosylated with primary and secondary alcohols. A benzylidene mannose donor resulted in the preferential formation of the *β*-anomer (Scheme 49b), whereas with 2-*O*-benzoyl-mannose the expected *α*-anomer was obtained (Scheme 49c). The proposed reaction mechanism involved the addition of glycoside hemiacetal to cyclic phosphonium anhydride, generating a mixture of anomeric intermediates. Subsequent addition of the alcohol acceptor afforded glycoside through direct displacement of the cyclic phosphonium ion. In the presence of a C2-participating substituent, the nucleophilic attack was directed by neighboring group participation. With acyclic phosphonium anhydrides, the phosphonium ion intermediate generated itself could undergo competitive reaction with the acceptor molecule, which could lead to the formation of a dimer product. Other sulfur-, nitrogen-, and carbon-based nucleophiles were also tried to carry out glycosylation under the standard conditions.

#### 2.9.2. Photochemical Synthesis

**Scheme 50:**
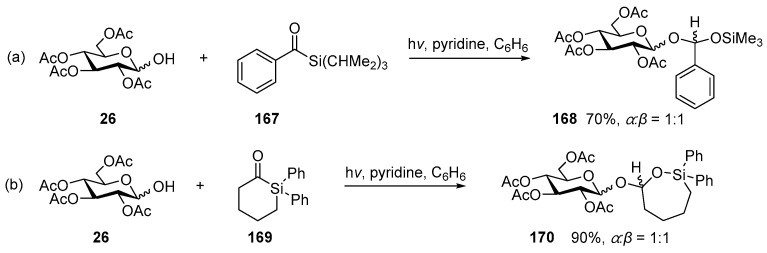
(**a**) Synthesis of siloxyacetal glycosides using acyclic acylsilane. (**b**) Synthesis of siloxyacetal glycoside using cyclic acylsilane.

The Svarovsky group reported a method for photochemical synthesis of siloxyacetal glycosides [76]. The photoreaction of peracetylated glucopyranosides **26** with acylsilanes **167** in the presence of catalytical amount of pyridine in anhydrous benzene afforded the desired glycosides **168** in good yields with inseparable anomeric mixtures (Scheme 50a). According to the proposed mechanism, the [1,2]-silyl shift of the acylsilanes formed highly reactive siloxycarbenes upon light irradiation and the resulting carbenes underwent intramolecular insertion into RO-H bonds of sugar substrates. The cyclic acylsilane substrates **169** were found to be much more efficient than the acyclic ones due to a higher tendency to form siloxycarbenes and gave a higher yield of adducts as a 1:1 mixture of diastereomers (Scheme 50b).

#### 2.9.3. Addition-Elimination (AE)

The application of *O*-linked aglycones in glycosidic linkage is essential in modern science. In carbohydrate chemistry, the oxidative addition and elimination reaction can be used for arylation of sugar molecules with aglycones. This reaction happens between a strong nucleophile like glycosyl oxide and an electrophilic arylating agent such as diaryliodonium salts. The mechanism for arylation involves the generation of glycosyl oxide as a nucleophile under basic conditions to react with the electrophilic iodine center of diaryliodonium salt followed by ligand exchange at the iodine center, giving a T-shaped intermediate, subsequent ligand coupling, or reductive elimination forms of *O*-Ar linkage.

**Scheme 51:**
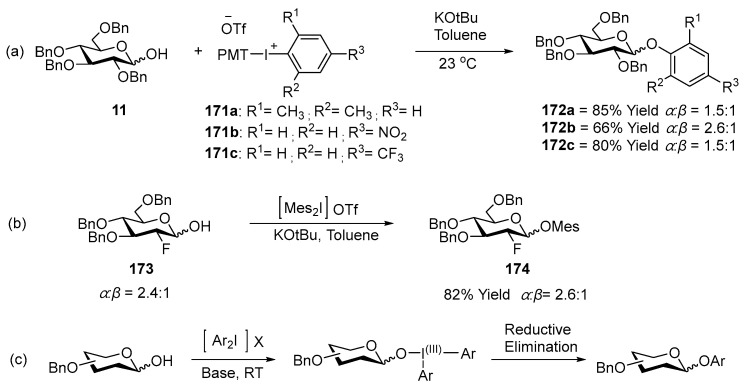
(**a**) Direct anomeric *O*-arylation of glycosyl hemiacetal with diaryliodonium reagents. (**b**) *O*-arylation of fluoro-substituted hemiacetal. (**c**) Proposed reaction mechanism.

Diaryliodonium salts are effective and multipurpose electrophilic arylating agents that have been used to arylate a wide range of nucleophiles. Steric factors are critical as manifested in the reaction with alcohol. Primary alcohols react well with these salts, while secondary alcohols have some limitations and tertiary alcohols are rarely used [77]. One of the advantages of these arylating reagents in glycosylation is that they do not require the pre-functionalization/activation sequence. Gilmour and coworkers investigated the potential of aryl iodonium salts for the arylation of inactivated hemiacetal sugars [78]. When reacting diphenyliodonium derivatives **171** with *O*-benzylated-D-glucopyranose **11** in the presence of *t*-BuOK in toluene, the predominate product was the glycoside with *α*-configuration (Scheme 51a).

When protected glucopyranose and furanose hemiacetals were reacted with a variety of symmetric and asymmetric iodine salts containing a 2,4,6-trimethoxyphenyl (TMP) group as an unexchangeable aryl component (**171a**, **171b**, **171c**), a variety of alkyl-, nitro-, and trifluoromethyl-substituted O-aryl glycosides (**172a**, **172b**, **172c**) with dominant *α*-configuration were produced. When the reactions were carried out on glycosyl hemiacetals carrying 2-fluoro substituents **173**, in the substrates in which the equilibrium between anomers favored the *α*-hemiacetal due to the anomeric effect, the *α/β* ratios of the products **17**4 resembled those of the starting hemiacetals. This observation showed that the *O*-arylation of glycosyl hemiacetals with diaryliodonium salts is a stereo-retentive process with no significant epimerization of the starting materials or the I(III) intermediates (Scheme 51c).

**Scheme 52:**
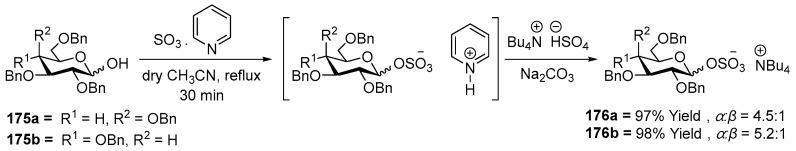
Preparation of anomeric sulfate donors.

The Imperio group reported a method for the synthesis of glycosyl sulfate donors by using SO_3_-pyridine complex [79]. The treatment of glucose or galactose hemiacetals **175** with SO_3_-pyridine complex in acetonitrile under reflux afforded the corresponding anomeric sulfates **176** in good yields as an anomeric mixture with predominant *α*-anomer (Scheme 52). During the work-up, the pyridinium counterion of the sulfate was replaced with tetrabutylammonium to improve solubility in organic solvents and to eliminate any weak proton source associated with the pyridinium ion. The resulted anomeric sulfates were used as glycosyl donors in the glycosylation reaction.

**Scheme 53:**
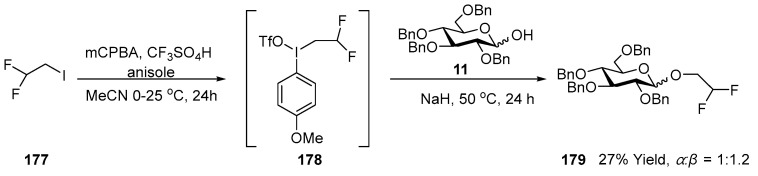
Fluoroethylation of benzylated D-glucose.

Recently, O’Duill and colleagues reported a method for electrophilic 2,2-difluoroethylation of different nucleophilic species like thiol, amine, and alcohols, including hemiacetal [80]. (2,2-Difluoroethyl)(4-methoxyphenyl)iodonium triflate reagent **178** was synthesized from commercially available 1,1-difluoro-2-iodoethane **177**. When the anomeric OH of benzyl-protected D-glucopyranose **11** was deprotonated in the presence of NaH, it could serve as a nucleophile to attack **178** and afforded the glycoside **179** as a mixture of anomers in low yield and low selectivity (Scheme 53). The proposed reaction mechanism involved nucleophilic attack followed by ligand exchange and subsequent reductive elimination, which formed a C-O bond between sugar and difluoroethane.

## 3. Reaction of Anomeric Hydroxyl on Unprotected Sugars

Direct conversion of the anomeric hydroxyl group from alcoholic hydroxyl groups requires the differentiation based on the higher acidity of acetal hydroxyl. In the presence of a proper base, it can be deprotonated and the resulting alkoxide is more nucleophilic to allow selective reaction with electrophiles. Aside from the regioselectivity, the compatibility of reagents with other hydroxyl groups, as well as solubilizing unprotected sugar, are also challenges in the anomeric hydroxyl conversions. The solvents are limited protic solvents like water and alcohol, or polar aprotic solvents such as DMF or acetonitrile.

### 3.1. Nucleophilic Substitution

2-Chloro-1,3-dimethylimidazolinium chloride (DMC), also known as Shoda’s reagent, was originally used as a coupling reagent in peptide synthesis and was reported to be extraordinarily electrophile to react with anomeric hydroxyl group in basic conditions. The glycosyl imidazolinium intermediate resulting from the addition–elimination type of reaction was subsequently converted into various glycosides and glycoconjugates by reacting with nucleophiles such as thiol, phenol, and azide. Excellent *β*-selectivity was observed in most of the reactions and involvement of 1,2-anhydro sugar was proposed to mechanistically explain the stereo outcome. However, substantial proof of the mechanism is hard to attain due to the complexity of the reaction mixture, and the involvement of glycosyl chloride could not be ruled out. It is reported that replacement of the C2-chloro in DMC with azide or thiolate can produce the desired product efficiently, and an intramolecular displacement of an immidazolinium leaving group was proposed Nonetheless, this protection-free and stereoselective reaction has attracted much attention and extensive applications of DMC in carbohydrate chemistry have been reported and reviewed by Faribanks et al. [81]. Recent studies are reviewed here to highlight the latest advancements regarding this reaction.

**Scheme 54:**
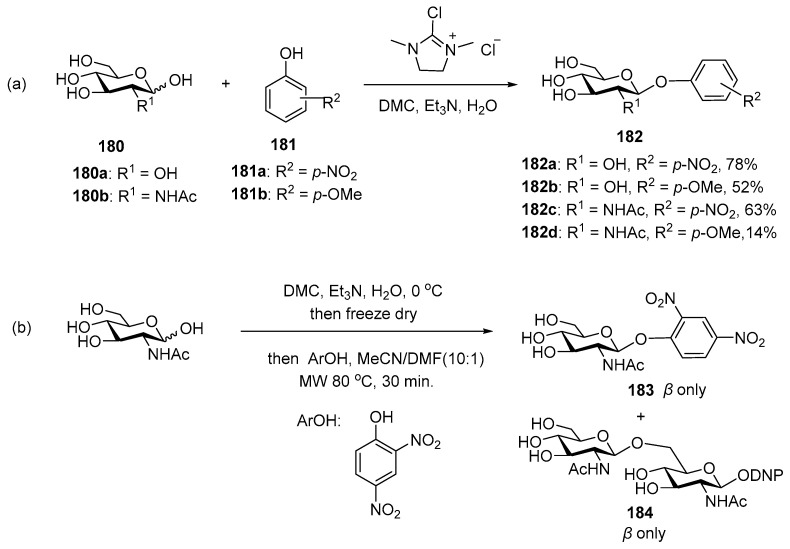
(**a**) DMC mediated nucleophilic substitution at anomeric carbon of unprotected sugars. (**b**) Disaccharide formation along with DNP glucoside during DMC mediated nucleophilic substitution.

In 2020, the Fairbanks group reported the activation of unprotected sugars in aqueous solution using DMC to synthesize *p*-nitrophenol glycoside (*p*-NP-glycoside) [82]. The treatment of reducing aldoses **180** with DMC in the presence of an excess amount triethylamine and *p*-nitrophenol **181** in water at −10 °C afforded the desired glycosides **182** with predominant *β*-anomer (Scheme 54a). For 2-acetamido sugars, the reaction was less efficient with phenols other than *p*- or *o*-nitrophenol due to the formation of an oxazoline intermediate. A more practical approach for forming aryl glycosides from 2-acetamido sugars was studied to generate oxazoline intermediates followed by solvent removal via freeze drying. The crude reaction product obtained was then treated with a phenol in a polar aprotic solvent under heat, which resulted in the formation of aryl glycoside in moderate yields. However, this method only worked for relatively acidic phenols.

In 2022, the same group continued their research on unprotected 2-acetamido sugars [83], and they noted that in the reaction where oxazoline was formed from 2-acetamido sugars when treated with DMC/Et_3_N in water, freeze drying the crude product, followed by dissolution in an appropriate anhydrous aprotic solvent and then addition of phenol, unexpectedly led to disaccharide side products **184** along with DNP-glycoside **183** (Scheme 54b). It was concluded that in addition to directly reacting with the oxazoline to form the desired glycoside, di-nitrophenol also catalyzed the self-reaction of GlcNAc oxazoline, resulting in disaccharide formation. This was an acid-catalyzed reaction, which was further studied to increase the yield of these disaccharide byproducts.

The Jayaraman group reported the alkylation and acylation of unprotected sugar in aqueous solution with the help of a 4-pyridoneimine base [84]. They demonstrated that 4-pyridoneimine deprotonates the anomeric hydroxyl group and the obtained anion reacts with acceptors to selectively achieve the functionalization at the anomeric carbon. Different acceptor molecules were used, for instance allyl, benzyl, propargyl halides, benzoic, and phthalic anhydrides, for mono-*O*-alkylation and acylation at the anomeric carbon.

**Scheme 55:**
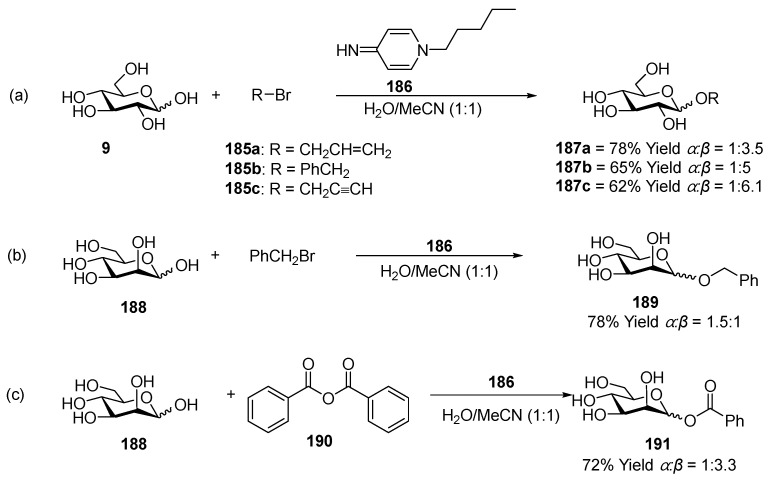
(**a**) Pyridoneimine-mediated glycosylation of glucose. (**b**) Pyridoneimine-mediated glycosylation of mannose. (**c**) Pyridoneimine-mediated glycosylation of mannose using benzoic or phthalic anhydrides.

The treatment of unprotected sugar **9** with alkyl and acyl bromide alkylating agents **185** in the presence of 4-pyridoneimine **186** as the base afforded *O*-glycosides **187** in moderate to good yields with a 1,2-*trans*-orientated product as the major anomer (Scheme 55a). The reactions with allyl bromide yielded allyl glycosides in relatively higher yield, followed by benzyl and propargyl glycosides. As compared to monosaccharide sugars, disaccharides afforded higher yields of glycosides. For mannose **188**, the corresponding glycosides were obtained with a substantial proportion of the *β*-anomer, which demonstrated a greater tendency toward the formation of the 1,2-*cis*-glycoside. Glycosides were predominantly formed as pyranosides, except for arabinose and ribose, which yielded both allyl pyranosides and furanosides. For arabinose, the glycosides were primarily pyranosides, with 1,2-*cis* being the major product, suggesting that the hemiacetal anion in the *β*-anomeric form is likely more reactive than the *α*-anomeric anion. When the benzoic or phthalic anhydrides were used as the alkylating agent, the reaction afforded the anomeric mono-*O*-acylated sugars **191** in moderate to good yields with predominant *β*-anomer (Scheme 55c). Again, disaccharides gave higher yields of *O*-acylated products as compared to monosaccharides.

This methodology solved the problem of multi-step protection and deprotections of sugars required for the functionalization of anomeric carbon. The aqueous solution conditions used here also helped in addressing the challenges of insolubility of unprotected sugars in most of the organic solvents.

**Scheme 56:**
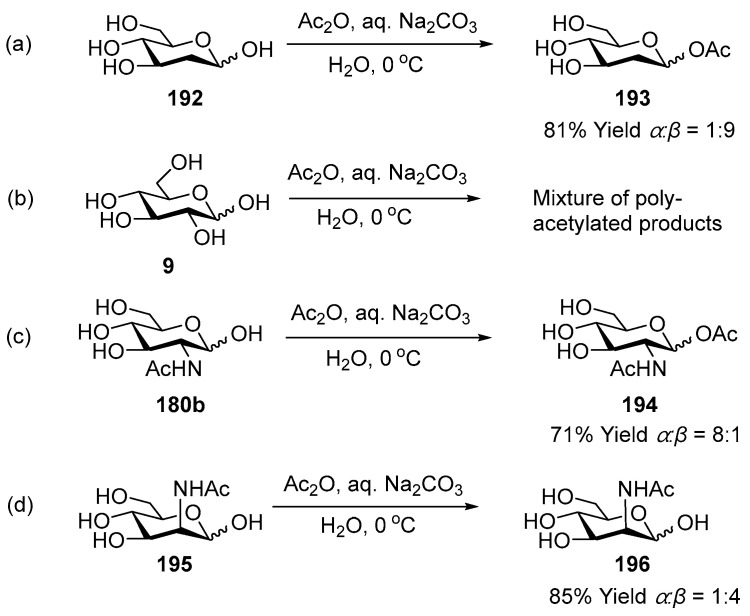
(**a**) Acetylation of unprotected 2-deoxy glucose using acetic anhydride. (**b**) Acetylation of glucose using acetic anhydride. (**c**) Acetylation of 2-acetomido glucose in presence of acetic anhydride. (**d**) Acetylation of 2-acetomido mannose in presence of acetic anhydride.

In 2023, the Fairbanks group reported a method for selective acetylation of the anomeric hydroxyl of unprotected sugar under mild basic conditions in the presence of acetic anhydride [85]. The treatment of sugar with acetic anhydride in the presence of Na_2_CO_3_ in aqueous solution at 0 °C afforded the desired glycosides as a mixture of anomers (Scheme 56). Acetylation of glucose **9** led to the formation of multiple products along with di- and polyacetylation products and unreacted glucose (Scheme 56b), with only a <5% yield of *β*-glycosyl acetate. The formation of multiple products was explained due to the migration of 1-*O*-acetate to the 2-position, and this migration resulted in the second and then third acetylation at the anomeric hydroxyl group. The NMR studies showed that the *α*-anomer of acetylated glucose was not stable under the reaction conditions and reacted rapidly via migration of the anomeric acetate to the 2-position to produce a material that could undergo further acetylation. In the case of mannose, it existed as the *α*-anomer in aqueous solution and the acetylation reaction gave *α*-acetate as the major product. There was insignificant formation of *β*-acetate, which could migrate to the 2-position, suggesting that anomerization competes with acetylation. When 2-deoxy glucose **192** was used as a substrate, the reaction yielded the corresponding glycosyl acetate **193** as a mixture of anomers in good yield (Scheme 56a), and 2-acetamido sugars **180b** gave acetylated products **194** with predominant *α*-configuration (Scheme 56c). An exception was observed with 2-acetamido mannose **195**, which formed products with predominant *β*-configuration (Scheme 56d). When disaccharides were investigated as substrates, corresponding disaccharide acetate was formed with predominant *α*-anomer.

**Scheme 57:**
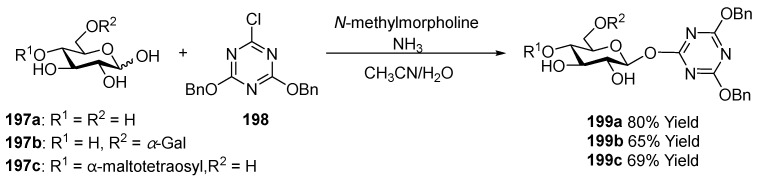
Synthesis of DBT glycosyl donors using *N*-methylmorpholine.

Shoda and coworkers reported a method for the synthesis of unprotected glycosyl donors bearing 2-chloro-4,6-dibenzyloxy-1,3,5-triazine (CDBT) as the leaving group [86]. Triazinyl glycoside derivatives are unique glycosyl donors with reactivity that is tunable by changing the functional groups at the 4 and 6 positions of the triazine ring through the S*_N_*Ar reaction. Dibenzyloxy-1,3,5-triazin-2-yl glycosides (DAT-glycosides) were prepared directly via alkaline nucleophilic substitution of unprotected sugars. The treatment of unprotected sugar **197** with CDBT **198** in the presence of *N*-methylmorpholine (NMM) and ammonia in aqueous media afforded the corresponding glycoside **199** with predominant *β*-configuration (Scheme 57). The results demonstrated that the *β*-anomer of hemiacetal selectively attacked the 2-position of the triazine ring to replace the chloride leaving group. The *β*-selectivity was due to the higher nucleophilicity of the *β*-hemiacetal oxide compared to the equilibrating *α* counterpart in water. Additionally, DBT-*β*-glycosides of larger oligosaccharides, such as melibiose and maltopentaose, were successfully synthesized without disrupting internal glycosidic bonds, whereas DMT-*α*-glycoside was obtained from D-mannose with an axial hydroxy group at the 2-position.

In 2023, Shoda’s group synthesized DBT glycosyl donors from unprotected hemiacetals using DABCO as the base, resulting in improved yields of DBT-β-glycosides compared to the *N*-methylmorpholine (NMM)/aqueous ammonia system [87]. For 2-acetamido sugars, NMM combined with the less basic 2,6-lutidine was used instead of DABCO. The treatment of D-galactose **200a** with CDBT in the presence of DABCO afforded DBT-*β*-glycosides **202a**. A trace amount of *α*-glycoside was observed at early stages of the reaction, which was further converted to 1,2-disubstituted derivative (1,2-(DBT)2-*α*-Gal) because the H-bond interaction between the nitrogen atom on the triazine ring enhanced the nucleophilicity of the hydroxy group at the 2-position of DBT-*α*-Gal, leading to the formation of the 1,2-disubstituted product. The yield of DBT-*β*-Gal **202a** was found to be less than that of DBT-*β*-Glc **202b** because of the instability of the latter in aqueous solution at room temperature.

**Scheme 58:**
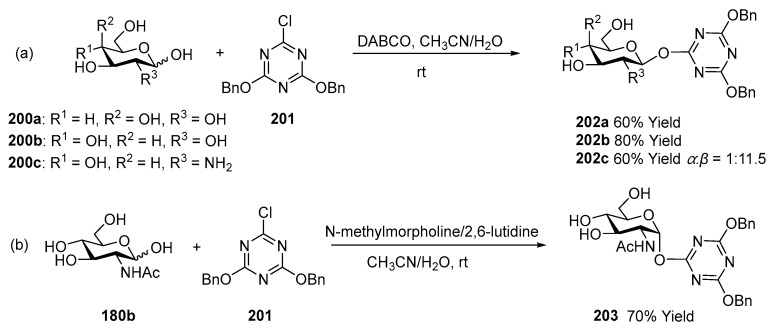
(**a**) Synthesis of DBT glycosyl donors using DABCO. (**b**) Synthesis of DBT glycosyl donors using *N*-methylmorpholine (NMM)/aqueous ammonia.

In the case of D-mannose, *α*-glycoside was obtained as the product along with some byproducts such as 1,2-(diDBT)-2-mannoside. The DBT glycosides from 2-deoxy sugars were not able to be isolated due to their greater susceptibility to hydrolysis. The reaction of D-glucosamine (GlcN) **200c** with CDBT afforded *β*-glycoside as the predominant product in moderate yield (Scheme 58a). The DBT derivative of 2-acetamido sugars **180b** was synthesized using *N*-methylmorpholine/2,6-lutidine system and afforded the desired glycosides with predominant *α*-configuration (Scheme 58b). The DBT donors synthesized was then used for glycosylation with various alcohols under mild hydrogenolytic conditions, which stereoselectively afforded corresponding alkyl glycosides in good yields.

**Scheme 59:**
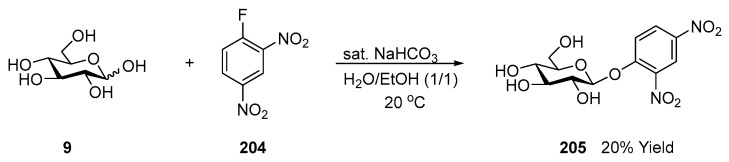
Synthesis of 2,4-dinitrophenyl glycosides.

The Pendas group reported a method to synthesize unprotected 2,4-dinitrophenyl glycosides in a single step from mono- and disaccharides [88]. The treatment of glucose **9** with 1-fluoro-2,4-dinitrophenol **204** in the presence of sodium bicarbonate solution afforded predominant 1,2-*trans* glycosides **205**, which are a kinetically controlled product (Scheme 59). Different sugar substrates like galactose, mannose, arabinose, xylose, and maltose were glycosylated successfully.

### 3.2. Nucleophilic Addition

**Scheme 60:**
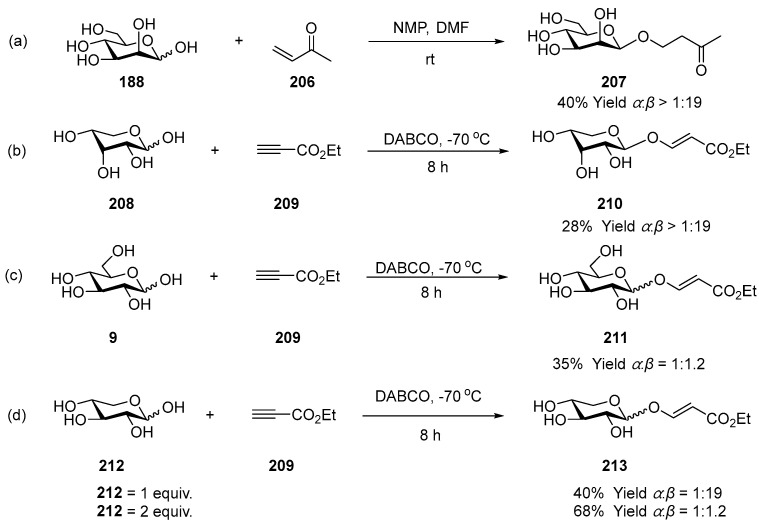
(**a**) Michael addition of anomeric OH of mannose with methyl vinyl ketone. (**b**) Michael addition of anomeric OH of ribose with ethyl propiolate. (**c**) Michael addition of anomeric OH of glucose with ethyl propiolate. (**d**) Michael addition of anomeric OH of xylose with ethyl propiolate.

In 2014, Mahrwald and coworkers reported a base-catalyzed addition of unprotected and inactivated carbohydrates to activate alkenes and alkynes via Michael addition [89]. The reaction was initiated by deprotonation of the anomeric hydroxyl group of free sugar and the resulting oxide would act as a nucleophile to attack an electron-deficient *α*,*β*-unsaturated carbonyl compound. The treatment of different hexoses **188** with methyl vinyl ketone **206** in the presence of *N*-methylpyrrolidine (NMP) in DMF at room temperature afforded desired glycosides **207** with predominant *β*-anomer (Scheme 60a). When activated, a terminal alkyne substrate like ethyl propiolate **209** was reacted with ribose **208** in the presence of DABCO at −70 °C, and selective formation of the corresponding enol riboside **210** was isolated in very low yield (Scheme 60b). Different hexose and pentose sugars successfully underwent addition with alkynes with a high degree of *β*-stereoselectivity. An exception was observed in glucose **9** when enol glucoside **211** was isolated as a mixture of anomers, which indicated a thermodynamically controlled reaction (Scheme 60c). Internal alkynes were also conjugated with ribose and xylose, albeit at a higher temperature (−40 °C). The yields of products were improved by increasing the amount of starting carbohydrates. However, variations in stereoselectivity were observed under these conditions. For instance, when 1 equivalent of xylose **212** was used, the corresponding product was obtained with a 40% yield and a high diastereomeric ratio (dr > 1:19). Increasing the xylose to 2 equivalents raised the yield to 68%, but the stereoselectivity decreased, resulting in enol xyloside with a diastereomeric ratio of 1:1.2 (*α*:*β*) (Scheme 60d).

The relatively higher pKa value (11–13) of lactols in mono- or disaccharides distinguishes itself from alcoholic hydroxyls, which have pKa values of 16–18 [90,91,92]. Recently, the Jayaraman group identified 4-pyridoneimine, which has pKa of ~15 in aq. solutions to be used as a base for the selective deprotonation of hemiacetal [84]. This base has been used for selective alkylations and acylations on free mono- and disaccharides.

**Scheme 61:**
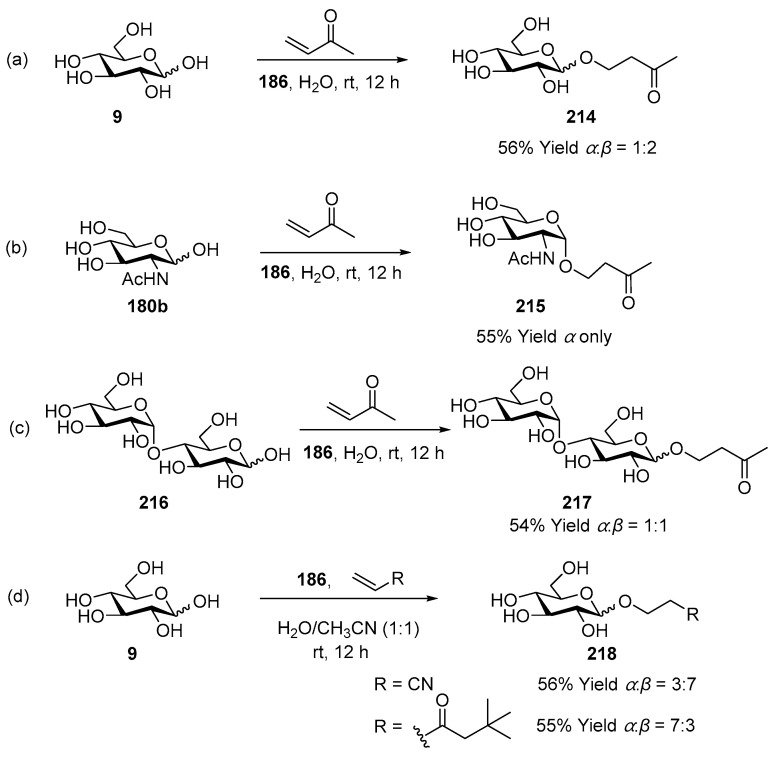
(**a**) Michael addition of anomeric OH to *α,β*-unsaturated synthons. (**b**) Nucleophilic addition of 2-acetomido glucose with methyl vinyl ketone. (**c**) Nucleophilic addition of maltose with methyl vinyl ketone. (**d**) Nucleophilic addition glucose with acrylonitrile and *tert*-butyl acrylate.

In 2022, Jayaraman and coworkers reported the pyridoneimine-catalyzed oxa-Michael addition of the protecting group of free mono- and disaccharides in aqueous solution [93]. The addition occurred site-selectively at the anomeric lactol, and the remaining hydroxy functionalities were unaffected. When an aqueous solution of D-glucose **9** was treated with methyl vinyl ketone in the presence of a pyridoneimine base at room temperature, it resulted in the formation of keto-glucopyranoside **214** as a mixture of *α*/*β*-anomer (Scheme 61a). The proposed mechanism of this reaction involved the deprotonation of the relatively acidic hemiacetal by pyridoneimine, which initiated a conjugate addition with methyl vinyl ketone and a byproduct 4-aminopyridinium ion. This addition produced an enolate, and a subsequent proton transfer assisted by 4-aminopyridinium ion completed the reaction. Additionally, pyridoneimine was suggested to tautomerize to its zwitterionic form in aqueous solutions, as previously reported by Pagani and coworkers [94].

The reaction of D-galactose and L-fucose pyranosides under these conditions afforded corresponding glycosides in moderate yield with predominant 1,2-*trans*-anomer, whereas D-mannose, L-rhamnose, and 2-deoxy-2-acetamido **180b** glucopyranoside afforded the 1,2-*cis*-anomer as the major product (Scheme 61b). In the case of disaccharides like maltose and lactose, the corresponding glycosides were obtained as an inseparable mixture of *α*/*β*-anomers (Scheme 61c). Pentose monosaccharides like ribose and arabinose afforded glycosides in moderate yields as *α*/*β* mixtures.

Aside from methyl vinyl ketone, acrylonitrile and *tert*-butyl acrylate were utilized as Michael acceptors. Reactions of D-glucose with acrylonitrile and *tert*-butyl acrylate were conducted in the presence of pyridoneimine (Scheme 61d), and a mixed solvent system of H₂O/MeCN (1:1) was employed to address the poor solubility of the reagents in water. The reactions proceeded smoothly, yielding cyanoethyl glycoside as an inseparable mixture of *α*/*β* anomers. Similarly, the reaction with *tert*-butyl acrylate produced glycoside in moderate yield with an *α*/*β*-anomers. The obtained keto-glucopyranosides were conjugated further with amino acids through reductive amination to form different kinds of derivatives, for instance, cinnamoyl derivative.

### 3.3. Mistsunobu Reaction and Appel Reaction

**Scheme 62:**
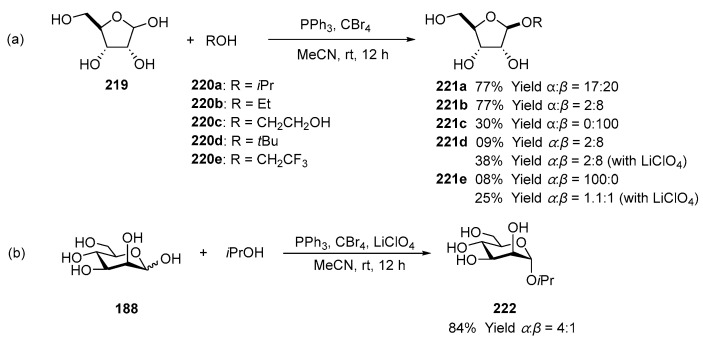
(**a**) Glycosylation of cyclic hemiacetals with aglycones. (**b**) Glycosylation of mannose with isopropanol in presence of LiClO_4_.

Also due to the higher acidity, the Mitsunobu reaction and Appel reaction can selectively react on the anomeric hydroxyl group in the presence of an alcoholic hydroxyl group. In 2013, the Mahrwald group reported an organocatalyzed direct glycosylation of free and inactivated sugars to form acetals of enolizable aldehydes in the presence of catalytic amounts of triphenylphosphine (TPP) and substituted tetrahalomethanes at room temperature under neutral conditions [95]. The reaction between unprotected ribose **219** and isopropanol resulted in the formation of isopropylriboside **221a** in good yields with high *β*-selectivity (Scheme 62a). Mechanistically, TPP and tetrabromomethane generated electrophilic phosphonium intermediate, which activated the anomeric hydroxyl group of sugar by forming a good leaving group. The nucleophilic isopropanol displaced the leaving group and afforded glycoside. To increase the yield of these reactions, lithium perchlorate salt was used, which resulted in direct catalytic glycosylation of free sugars.

Different carbohydrate substrates like deoxyribose, arabinose, xylose, and lyxose afforded glycosides in low-to-high yields with predominate *β*-anomer. Mannose resulted in the formation of thermodynamically favored *α*-configured isopropyl glycoside as the main product (Scheme 62b). Different nucleophilic alcohol substates like ethyl alcohol, trifluoroethanol, *tert*-butanol, and diols (**220b**, **d**, **e**) were used for the reaction with ribose. The corresponding ribosides were isolated in moderate-to-high yields, except for the reactions of ribose with *tert*-butanol and trifluoroethanol, which resulted in low yields due to the electronic and steric effects of the aglycones; however, the yields could be improved by using lithium perchlorate (Scheme 62a). Moreover, a moderate-to-high *β*-selectivity was observed in these glycosylation reactions, with excellent diastereoselectivities achieved when aglycones containing oxygen (**220c**) were used. By reacting ribose with Cbz-protected serine methyl ester under standard conditions, the utility of this method was demonstrated.

**Scheme 63:**
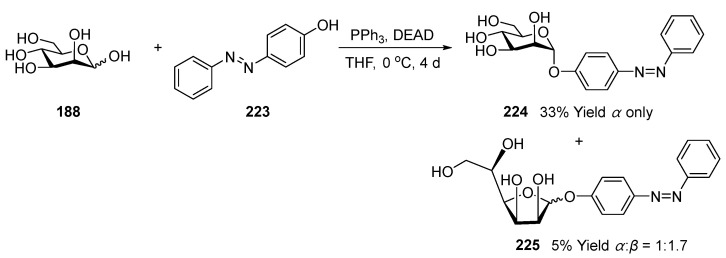
Synthesis of *α*-mannopyranosyl hydroxyazobenzene donors via Mitsunobu protocol.

In 2015, Lindhorst and colleagues reported a method for the synthesis of *α*-mannopyranosyl hydroxyazobenzene donors using the Mitsunobu reaction [96]. The treatment of unprotected mannose sugar with *p-*hydroxyazobenzene afforded the desired aryl *α*-mannopyranoside in moderate yield with a slight anomeric mixture of mannofuranosides as side products (Scheme 63).

**Scheme 64:**
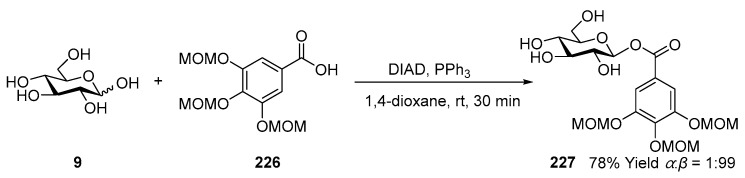
Stereoselective glycosylation of unprotected glucose to give *β*-glucosyl benzoic acid.

In the same year, Kawabata and coworkers performed the Mitsunobu reaction on an unprotected and inactivated sugar as the initial step in the total synthesis of two ellagitannins [97]. When free glucose **9** was treated with gallic acid trimethoxymethyl ether **226** in the presence of DIAD, PPh_3_, and 1,4-dioxane, it afforded *β*-glucosyl benzoic acid in good yield with high stereoselectivity (Scheme 64). The resulted glycoside was further used in the synthesis of both strictinin and telligran. In 2021, the Kawabat group also employed this method as an initial step in the synthesis of punicafolin and macaranganin from D-glucose [98].

**Scheme 65:**
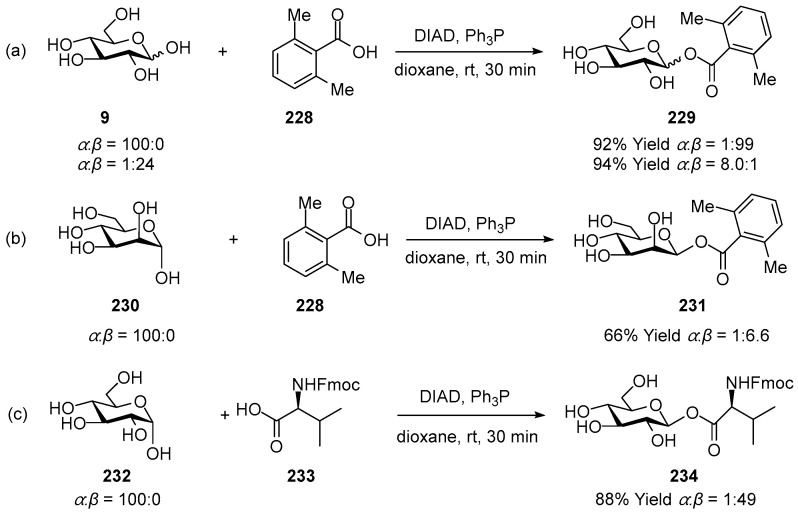
(**a**) Stereoselective glycosylation of benzoic acid with glucose. (**b**) Stereoselective glycosylation of benzoic acid with mannose. (**c**) Stereoselective glycosylation of chiral center containing acids with glucose.

In 2020, the Kawabata group reported a highly stereoselective glycosylation of unprotected D-glucose with benzoic acid under Mitsunobu conditions in dioxane via the S*_N_*2 mechanism, whereas in DMF glycosylation was found to procced via the non-stereoselective S*_N_*1 mechanism [99]. When unprotected *α-*glucose **9** was treated with benzoic acid derivative **228** in the presence of DIAD, PPh_3_, and solvent (dioxane or DMF), it afforded anomeric mixtures of glycoside (Scheme 65a). The *β*-selective glycosylation was due to the displacement of the oxyphosphonium ion with an *α*-configuration by the benzoate anion via an S*_N_*2 mechanism. In the S*_N_*1 mechanism, the *β*-selectivity was explained by similar displacement via a contact ion pair-like intermediate, where the *α*-face of the oxonium cation was shielded by the oxyphosphonium zwitterion in the polar solvent, i.e., dioxane. In contrast, the non-stereoselective glycosylation in DMF was attributed to the involvement of a solvent-separated ion pair.

Different sugar substrates reacted with 2,6-dimethyl benzoic acid to form the corresponding glycosides in good yields with inversion of configuration. The glycosylation of unprotected α-D-mannose also proceeded via an S*_N_*2 mechanism and preferably yielded the less-accessible 1,2-*cis*-mannoside (Scheme 65b). The scope of sugars was explored to find that aliphatic and aromatic carboxylic acids with various functional groups were well tolerated, yielding the corresponding glycosides in high *β*-selectivity. Glycosylation of acids with an *α*-chiral center occurred without any epimerization at the chiral center (Scheme 65c). Phenol derivatives and imides were also glycosylated successfully. The application of this method was demonstrated in the formation of a drug–glucose conjugate under the standard conditions.

**Scheme 66:**
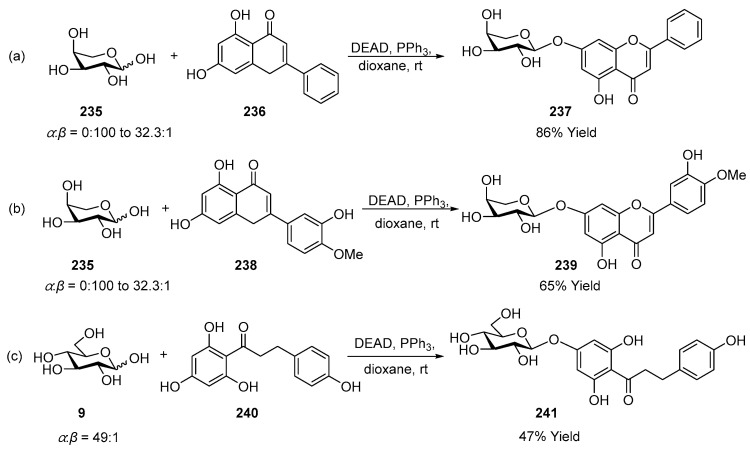
(**a**) Synthesis of flavonoid glycosides by Mitsunobu glycosylation of arabinose with chrysin. (**b**) Synthesis of flavonoid glycoside of arabinose by Mitsunobu glycosylation. (**c**) Synthesis of flavonoid glucosides by Mitsunobu glycosylation.

The Lu group reported a method for the synthesis of flavonoid *O*-glycosides from protecting-group-free pyranoses under Mitsunobu conditions [100]. This methodology was used in the preparation of a wide range of natural 7-flavonoid *O*-glycosides and their derivatives from commercially available chemicals in good-to-excellent yields with 1,2-*trans*-stereoselectivity. The treatment of unprotected pyranose sugars like arabinose, xylose, and lyxose with different flavonoids such as chrysin and phloretin as acceptors in the presence of DEAD and PPh_3_, in dioxane at room temperature, afforded the corresponding 1,2-*trans*-glycosylation product (Scheme 66a). The study demonstrated equal efficiency in glycosylation among various acceptors like naringenin and hesperetin, indicating that the presence of a bond between the 2- and 3-positions of the C ring, as well as hydroxyl groups on the 3′- or 4′-positions of the B ring, had negligible influence on the reaction (Scheme 66b). Furthermore, when phloretin, an open-chain flavonoid with four free phenolic hydroxyl groups, was used, the glycosylation reaction exhibited excellent stereoselectivity and regioselectivity, despite moderate yields (Scheme 66c). The high regioselectivity among different types of hydroxyl groups of the acceptors was probably due to different acidity.

### 3.4. Phosphorylation

**Scheme 67:**
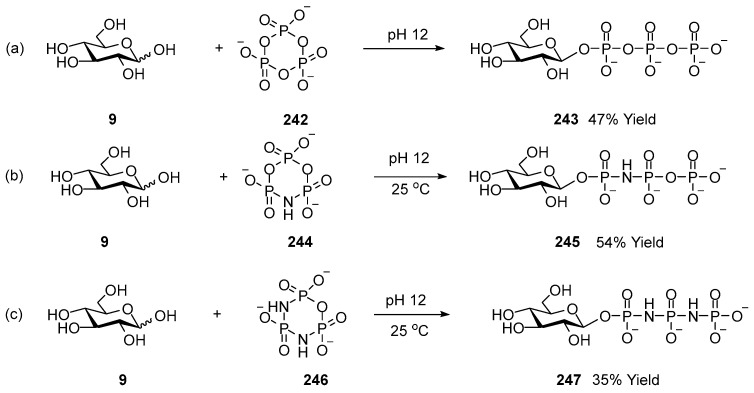
(**a**) Phosphorylation of glucose using cyclo-triphosphate (P_3m_). (**b**) Phosphorylation of glucose using MCTP. (**c**) Phosphorylation of glucose using DCTP.

The Tsuhako group reported a method for phosphorylation of D-glucopyranose **205** by using inorganic cyclo-triphosphate (P_3m_) **242** as the phosphorylating agent [101]. When glucopyranose was treated with cyclo-triphosphate at pH of 12, it afforded *β*-D-glucopyranosyl 1-triphosphate **243** in moderate yields (Scheme 67a). In 2006, the Tsuhako group reported the phosphorylation of glucose derivatives using inorganic monoimido-cyclotriphosphate (MCTP, Na_3_P_3_O_8_NH) in aqueous solution [102]. The treatment of D-glucose **9** with MCTP **244** at pH of 12 afforded 1-*O*-diphosphoramidophosphono-D-glucose **245** in moderate yield with *β*-selectivity (Scheme 67b). Different sugar substrates like D-glucuronic acid, 2-deoxy-D-glucose, and D-galactose were applied to this methodology and generated corresponding glycosyl phosphates with *β*-selectivity. For D-mannose and D-allose, several phosphorylated products were obtained, and the main products were 1-*O*-diphosphoramidophosphono-*β*-D-aldoses. The reaction mechanism involved the attack of the anomeric hydroxyl group of sugars on the phosphorus atom of MCTP, resulting in the cleavage of a six-membered ring. It was also noted that the existence of hydrogen bonding between oxygen of MCTP and the OH group at the C-2 position may facilitate the attack on MCTP. It was proposed that the *α*-hydroxyl group of aldose can hardly approach MCTP due to the steric hindrance caused by the 1,2-*cis* conformation of the hydroxyl groups or the 1,3-diaxial interaction between H-3 and the monoimidotriphosphate group.

In 2007, the same group used inorganic diimido-cyclo-triphosphate (DCPT) as a phosphorylation agent for the phosphorylation of D-glucose and gluco-oligosaccharides [103]. As shown in Scheme 67c, treatment of glucose **9** with DCPT **246** in aqueous solution afforded phosphate glycoside with *β*-selectivity. Gluco-oligosaccharides like maltose also afforded the desired phosphorylated products as *β*-anomer. For *α*-cyclodextrin (*α*-CD), γ-cyclodextrin (γ-CD), or mono-6-*O*-*α*-maltosyl-*β*-cyclodextrin (mono-G2-*β*-CD), two phosphorylated products were obtained, and the main products were mono-2-*O*-diimidotriphosphoryl esters.

**Scheme 68:**
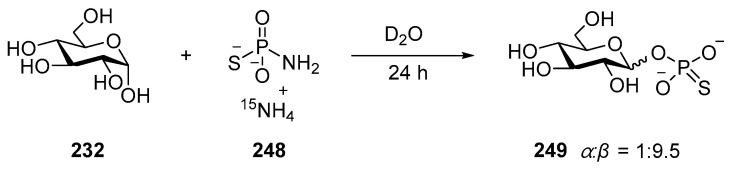
Phosphorylation of glucose using diammonium thiophosphoramidates.

Ruman and colleagues reported a method to synthesize ^15^*N*-isotope-enriched potassium and diammonium thiophosphoramidates and studied their reactivity towards different compounds [104]. When unprotected glucose **232** was thiophosphorylated with synthesized thiophosphoramidates **248**, glucose-1-thiophosphate was formed as a mixture of anomers with high *β*-selectivity (Scheme 68).

**Scheme 69:**
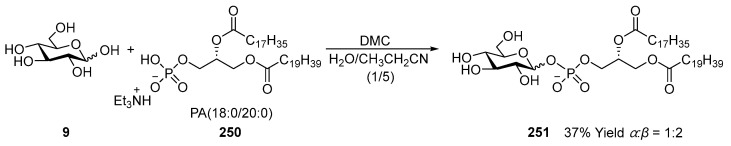
DMC-mediated phosphorylation.

The Matsuo group reported the 2-chloro-1,3-dimethylimidazolinium chloride (DMC) mediated one-pot synthesis of glycosyl phosphate using phospholipid derivative [105]. The treatment of glucose **9** with phosphatidic acid **250** in the presence of triethylamine and DMC afforded phosphatidyl glycoside **251** as a mixture of anomers with high *β*-selectivity in moderate yields (Scheme 69). A quadratic relationship was observed between the reaction yield and water content in the solvent system of H_2_O/C_2_H_5_CN, with the maximum yield achieved at water contents between 40 and 50%. In contrast, the stereoselectivity was significantly reduced in mixtures with less than 40% water content. When glucose and DMC were added stepwise to phosphatidic acid, an increase in the yield of the products was observed, indicating that DMC and the glycosyl donor were the primary limiting factors responsible for moderate yields. It is proposed that the reaction proceeds by the formation of a 1,2-anhydro sugar intermediate, followed by the nucleophilic attack of the acceptor nucleophile. Different phosphatidic acid acceptors were tolerated.

## 4. Conclusions

As the most abundant biomolecule, carbohydrates are ubiquitously involved in many fundamental biological processes and their significance is increasingly being acknowledged. Accordingly, chemical synthesis, especially glycosylation, is fast-growing. The conversion of the anomeric hydroxyl group of the hemiacetal is of great importance and the most challenging reaction in carbohydrate synthesis. The control of the stereoselectivity in this conversion requires advancement of our understanding of the properties of the hydroxyl groups, as well as the techniques for kinetic and thermodynamic manipulation of the reactivity. By summarizing the recent advancements in this field, this review aims to gather the information to facilitate the incubation of new methods for the regio- and stereoselective conversion of the anomeric hydroxyl group, and hence significantly change the feedstock of carbohydrate molecules to boost related biological, medicinal, and industrial research.

## Data Availability

Not applicable.
